# Novel *NUP98::ASH1L* Gene Fusion in Acute Myeloid Leukemia Detected by Optical Genome Mapping

**DOI:** 10.3390/cancers15112942

**Published:** 2023-05-27

**Authors:** Marco Tembrink, Wanda Maria Gerding, Stefan Wieczorek, Thomas Mika, Roland Schroers, Huu Phuc Nguyen, Deepak Ben Vangala, Verena Nilius-Eliliwi

**Affiliations:** 1 Department of Human Genetics, Ruhr-University Bochum, 44801 Bochum, Germany; marcotembrink@gmail.com (M.T.);; 2 MVZ Dr. Eberhard & Partner Dortmund GbR (ÜBAG), 44137 Dortmund, Germany; 3 Department of Medicine, Hematology and Oncology, Knappschaftskrankenhaus, Ruhr-University Bochum, 44892 Bochum, Germanyverena.nilius-eliliwi@rub.de (V.N.-E.)

**Keywords:** clonal evolution, rare variant pipeline, disease monitoring: structural variants, cytogenetics, karyotyping

## Abstract

**Simple Summary:**

Structural genetic variants, such as gene fusions, are frequently detected in hematologic neoplasias. Over the past few decades, these structural variants have gained clinical impact via their guidance of disease classification and risk stratification. *NUP98*-rearrangements are recurrent in different hematologic neoplasias, fusing the gene *NUP98* to a wide variety of partners. As they are often cytogenetically cryptic, other methodologies are required for the detection and identification of genetic fusion partners. Here, a previously unrecognized *NUP98::ASH1L* fusion was detected in a patient with acute myeloid leukemia with prior myelodysplastic/myeloproliferative neoplasia using a genome-wide approach through optical genome mapping. Using this technique, the clonal evolution of structural aberrations was detected during the early relapse after allogeneic stem cell transplantation. These findings give a putative explanation for the aggressive disease course and demonstrate the potential of this method for consecutive disease monitoring.

**Abstract:**

Optical genome mapping (OGM) recently has demonstrated the potential to improve genetic diagnostics in acute myeloid leukemia (AML). In this study, OGM was utilized as a tool for the detection of genome-wide structural variants and disease monitoring. A previously unrecognized *NUP98::ASH1L* fusion was detected in an adult patient with secondary AML. OGM identified the fusion of *NUP98* to *Absent, Small, or Homeotic-Like Histone Lysine Methyltransferase (ASH1L)* as result of a complex structural rearrangement between chromosomes 1 and 11. A pipeline for the measurement of rare structural variants (Rare Variant Pipeline, Bionano Genomics, San Diego, CA, USA) was used for detection. As *NUP98* and other fusions are relevant for disease classification, this demonstrates the necessity for methods such as OGM for cytogenetic diagnostics in AML. Furthermore, other structural variants showed discordant variant allele frequencies at different time points over the course of the disease and treatment pressure, indicating clonal evolution. These results support OGM to be a valuable tool for primary diagnostics in AML as well as longitudinal testing for disease monitoring and deepening our understanding of genetically heterogenous diseases.

## 1. Introduction

Recurrent genetic variants are highly relevant for the classification and risk stratification of patients with acute myeloid leukemia (AML) [1]. Apart from amplification-based techniques such as polymerase chain reaction (PCR) and sequencing, cytogenetic methods provide important information for prognostic stratification and therapeutic decisions [2,3]. Recurrent rearrangements at 11q15.4 involving the gene *Nucleoporin 98 and 96 precursor* (*NUP98*) are found in a variety of hematologic neoplasias, such as AML, myelodysplastic neoplasias (MDS), and T-cell acute lymphoblastic leukemia (T-ALL) [4,5]. The incidence of *NUP98*-rearrangements in AML seems to be higher in pediatric AML patients (~2.5–5%) compared to adult cohorts (~0.5–2.5%) [6,7,8,9,10]. *NUP98* fusions are associated with poor prognosis in pediatric patients because of chemotherapy resistance and a higher risk of relapse. In addition, the most common *NUP98* fusions were associated with an inferior outcome in adults and resistance to chemotherapy [11,12,13,14,15]. In adults, a clear correlation to prior treatments or other morphological findings has not been described so far. Evidence for the prognostic implications of rare *NUP98* fusions remains largely unclear due to the vast number of fusion partners and limited information available based on a small number of cases. Additional research is needed to determine their clinical significance [6,9,16,17]. From a clinical point of view, *NUP98* fusions have recently garnered attention, as they are now included in the latest versions of the broadly accepted classification guidelines for AML of the World Health Organization (WHO) and the European LeukemiaNet (ELN) [1,18,19,20]. 

Many *NUP98* rearrangements are cytogenetically cryptic because of the distal chromosome position of *NUP98* on 11p15.4 near the telomeric region. Therefore, other methods than chromosomal banding analysis (CBA) are necessary for deciphering these structural variants (SVs). A common example is the cryptic translocation t(5;11)(q35;p15), resulting in the gene fusion *NUP98::NSD1* [15,21]. Optical genome mapping (OGM) as a novel genome-wide amplification-free technique based on mapping of motif-labeled DNA molecules recently showed a high concordance with current standard genetic diagnostics in different hematologic malignancies, such as AML or MDS. Using high molecular weight DNA isolated from bone marrow aspirate and peripheral blood samples, the methodology was able to identify additional prognostic information, including *NUP98::NSD1* and, in one case, the rare fusion *NUP98::TNRC18* [22,23,24,25,26,27,28,29].

Here, we describe *NUP98::ASH1L* as a previously unrecognized *NUP98* gene fusion and discuss its possible pathogenetic contribution to early relapse after allogeneic hematopoietic stem cell transplantation (alloHSCT) in a patient with secondary AML and prior myelodysplastic/myeloproliferative neoplasia with ring sideroblasts and thrombocytosis (MDS/MPN-RS-T). *NUP98::ASH1L* was identified using OGM and its fusion transcript was further characterized via reverse-transcription PCR (RT-PCR). 

## 2. Materials and Methods

### 2.1. Patient Consent

At our tertiary care center, OGM has been implemented in parallel to routine clinical cytogenetic testing to evaluate its feasibility in acute leukemias, as described previously [23,28,30,31]. Written and informed consent was provided for patient participation in this study and for publication. The ethics committee of Ruhr-University Bochum approved of this work (No. 20-7063). 

### 2.2. Standard Diagnostics 

Cytomorphologic analyses, multiparameter flow cytometry and genetic donor chimerism testing were performed at University Hospital Knappschaftskrankenhaus Bochum. CBA according to the International System for Human Cytogenomic Nomenclature (ISCN) with a minimum of 20 metaphases analyzed using two unstimulated cell cultures per analysis [32,33] and the absolute quantitative PCR (qPCR) monitoring of a known *JAK2* V617F variant were performed at MVZ Dr. Eberhard & Partner, Dortmund, using accredited standard methods. An RT-PCR fusion panel (Mentype AMLplexQS, Biotype, Dresden, Germany) including ELN 2017 standard gene fusions (*RUNX1::RUNX1T1*, *CBFB::MYH11*, *PML::RARA*, *BCR::ABL1)* and 6 additional gene fusions *(DEK::NUP214*, *KMT2A::AFDN*, *KMT2A::MLLT3*, *KMT2A::ELL*, *NPM1::MLF1*, *PICALM::MLLT10*), standard ELN 2017 mutation analyses (*ASXL1*, *RUNX1*, *TP53*, *NPM1*, *CEBPA*, *IDH1/2*, *FLT3*-ITD/TKD), and histomorphological analyses were carried out by collaborating laboratories as part of routine clinical diagnostics. 

At the time of refractory anemia with ring sideroblasts and thrombocytosis (RARS-T, provisional entity) diagnosis in 2015, the Interphase-Fluorescence in situ hybridization (FISH) analyses of 200 nuclei using accredited routine diagnostic protocols, according to the manufacturers’ recommendations (Abbott: Vysis LSI 4q12 Tricolor Probe, Vysis ETV6 Dual Color Break Apart Rearrangement Probe; Kreatech: PDGFRB (5q32); Proximal FISH probe, FGFR1 (8p11) Break FISH probe; MetaSystems: XL ATM/TP53), and quantitative RT-PCR testing for *BCR::ABL1* transcripts (in-house test for *M-bcr*, *m-bcr*, and *µ-bcr*) were performed at MVZ Dr. Eberhard & Partner, Dortmund.

### 2.3. Optical Genome Mapping and Rare Variant Pipeline

OGM was performed as previously described using EDTA-anticoagulated bone marrow aspirate from three different time points (initial diagnosis, prior to alloHSCT, and at relapse) at the Department of Human Genetics, Ruhr-University Bochum [23,31]. Analyses were performed using Direct Labeling Enzyme-1 (DLE-1, Bionano Genomics, San Diego, USA) and OGM was carried out on a Saphyr instrument (Bionano Genomics, San Diego, USA). Rare Variant Pipeline (RVP) analyses were performed using the manufacturer’s software solutions (Bionano Access 1.7 and Bionano Solve 3.7).

Molecule quality parameters were evaluated following the manufacturer’s guidelines and were fulfilled for all three samples [34]. Effective 361-fold coverage at initial AML diagnosis, 629-fold coverage prior to alloHSCT, and 626-fold coverage at relapse compared to the Genome Reference Consortium Human Build 37 (GRCh37) were reached post-analysis. The OGM molecule quality parameters after RVP analysis, rare and confident SV output, confident copy number variant (CNV) output, and confident aneuploidy calls are depicted in Supplementary Tables S1–S4.

At AML diagnosis, a target throughput of 1500 Gigabases (Gb) was set to reach a minimum effective coverage of 300X for a VAF detection limit of approximately 5% in regard to SVs, according to the manufacturer [35]. For the assessment of residual disease prior to alloHSCT and at relapse, a higher target throughput of 2500 Gb was set, aiming to reach lower VAF detection limits.

Using the RVP pipeline, molecules with labeling difference compared to the labeling pattern of GRCh37 were clustered, generating SV calls. SVs were annotated automatically with a VAF calculated via the software using the label coverage of the SV call compared to the coverage of the reference. In addition, CNV calls were generated automatically via the software, comparing the coverage variation of the overall molecule labeling pattern to the GRCh37 reference, and the VAF of CNVs was calculated based on the fractional copy number of the CNV [36]. SV calls were annotated with population frequencies through comparison to 179 DLE-1-labeled healthy multiethnic controls, and SVs and CNVs were annotated and reported using a browser extensible data (BED) file, both provided by the manufacturer (hg19 known Canonical, Bionano Genomics, San Diego, USA). SVs and CNVs were assessed for confidence thresholds and overlap at regions of high genomic variance using default RVP SV and CNV masks in Bionano Access Version 1.7. Rare SVs were defined as SVs occurring in 1% or less of controls (confidence thresholds: insertion and deletion, 0; inversion, 0.7; duplication, -1; intra-chromosomal fusion, 0.05; inter-chromosomal translocation, 0.05; copy number variants, 0.99; aneuploidy, 0.95). In the case of duplicate SVs, the SV with the highest molecule count was output.

### 2.4. Reverse-Transcription Polymerase Chain Reaction

For the detection of the fusion transcript of *NUP98::ASH1L*, RNA was isolated and two independent targeted RT-PCR assays were performed as previously described [37]. Reverse primers for *ASH1L* (NM_018489) exon 8 (5′-gtttccactgccaaaggatatc-3′) and exon 10 (5′-caggtggtagcttcgtaacca-3′) were synthesized. A cDNA-specific PCR system was designed. A forward primer spanning *NUP98* (NM_139131) exons 12 and 13 (5’-gccccagtagctttgacaga-3’) was used. An *ASH1L* exon 6 reverse primer was used (5’-tcccttgggatgagagaaag-3’). Sequencing was performed on a 3500xL Genetic Analyzer (Applied Biosystems, Waltham, MA, USA), and sequences were aligned and visually inspected for *NUP98::ASH1L* product specificity (SeqMan software, DNAStar Inc., Madison, WI, USA). *NUP98::ASH1L* fusion breakpoint was determined using BLAT search (UCSC Genome Browser) [38].

## 3. Results

### 3.1. Case Description

A 57-year-old female patient was diagnosed with RARS-T according to the 4th edition of the World Health Organization (WHO 2008) in 2015 [39]. At that time, Interphase- FISH analyses excluded *PDGFRA*, *PDGFRB*, *FGFR1*, *ATM*, *ETV6*, or *TP53* abnormalities in 200 analyzed interphase nuclei. Three months earlier, a *JAK2* V617F-mutation had been detected and the presence of *BCR::ABL1* fusion transcripts was excluded in peripheral blood cells. Starting in 2015, the patient received treatment encompassing cytoreductive and anti-thrombotic agents (hydroxyurea and acetylsalicylic acid). 

**Figure 1:**
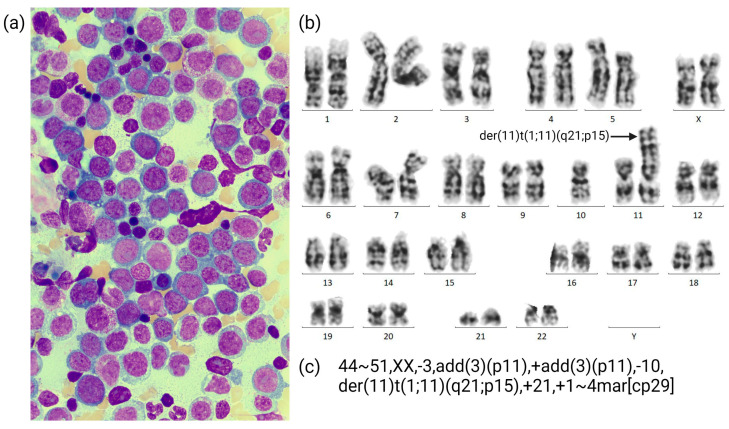
(**a**) Cytomorphology: bone marrow smear showing blast infiltration of 60–70% with minimal differentiation at diagnosis; (**b**) single karyogram at diagnosis; (**c**) karyotype at initial diagnosis.

Seven years later, at the age of 64 years, the patient presented with fatigue, progressive dyspnea, angina pectoris, and sinus tachycardia. Peripheral blood counts showed severe anemia with hemoglobin levels of 4.2 g/dL and thrombocytopenia (50/nL), but a normal leukocyte count (5.1/nL) without circulating blasts. Cytomorphology after bone marrow biopsy revealed a hypercellular bone marrow with a blast infiltration of 60–70% and minimal differentiation (Figure 1a). CBA showed a complex karyotype with a derivate chromosome 11, which was interpreted as a translocation t(1;11)(q21;p15), as well as several other chromosomal rearrangements (Figure 1b,c). Other molecular genetic variants were not identified via routine diagnostics.

In summary, this established the diagnosis of AML with myelodysplasia-related changes (AML-MRC), according to the WHO 2017 classification, which was current at the time. The prior RARS-T diagnosis was reclassified as MDS/MPN-RS-T [19]. Considering the complex karyotype with ≥3 aberrations in the absence of WHO 2017-designated recurrent translocations and inversions, the patient was grouped into the adverse risk group, according to the ELN 2017 recommendations [20]. 

The patient was treated with induction chemotherapy according to the ‘3 + 7’ protocol (days 1–3 daunorubicin 60 mg/m^2^, days 1–7 cytarabine 200 mg/m^2^) followed by a second induction cycle (days 1–2 daunorubicin 50 mg/m^2^, days 1–3 cytarabine 500 mg/m^2^ twice daily) achieving complete remission (CR) based on histomorphology, cytogenetics, and multiparameter flow cytometry. Consolidation treatment consisted of one cycle of cytarabine (2000 mg/m^2^ days 1–3). Subsequently, the patient underwent alloHSCT from an unrelated HLA-matched (10/10) female donor after receiving a reduced intensity conditioning (RIC) regimen (fludarabine, treosulfan). 

Although being in hematological CR after induction treatment, there was a relapse detected in molecular diagnostics directly prior to start of RIC (*JAK2* V617F 11.6%). After alloHSCT, the patient was in hematological CR. However, despite the quick tapering of immunosuppressants, molecular remission was not achieved. After an early hematological AML relapse as detected by donor/patient chimerism testing in bone marrow and peripheral blood samples on day +63 after alloHSCT, the patient received one course of a hypomethylating agent (azacytidine). Finally, the patient died on day +107 after alloHSCT due to AML relapse. The diagnostic results are summarized in Table 1.

### 3.2. OGM Findings

**Figure 2:**
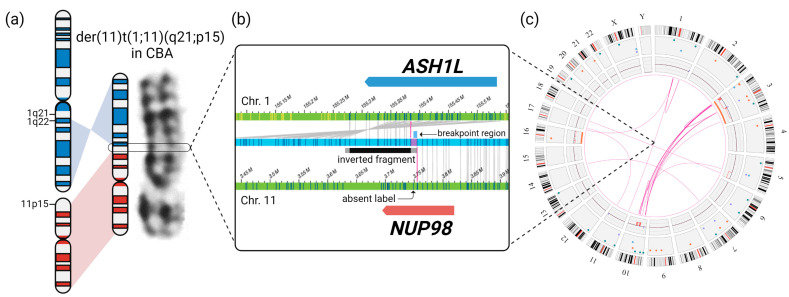
OGM resolved the underlying structural variations leading to a *NUP98::ASH1L* fusion (**a**) Derivative chromosome 11 rated as der(11)t(1;11)(q21;p15) in CBA and schematic rearrangement depicted as ideograms. (**b**) OGM molecule map (blue) where the labeling pattern indicates a rearrangement of chromosomes 1 and 11 (references in green), leading to a genomic *NUP98::ASH1L* gene fusion; for the visualized molecule map, an inter-chromosomal translocation and an inversion SV were called; the approximately 100 kb spanning inverted fragment is depicted in black (partly aligned to chromosome 1 labels) and grey (inversion break point regions); the breakpoint of the inter-chromosomal translocation utilized by the software (violet) was manually delimited to a smaller 8 kb region, as a label of chromosome 11 was absent in the molecule map (marked as “breakpoint region”). (**c**) Circos plot of OGM RVP at initial AML diagnosis filtered for confident SVs occurring in ≤1% of controls and confident CNVs. For Circos plots of consecutive OGM analyses see Appendix B Figure A1.

**Figure 4:**
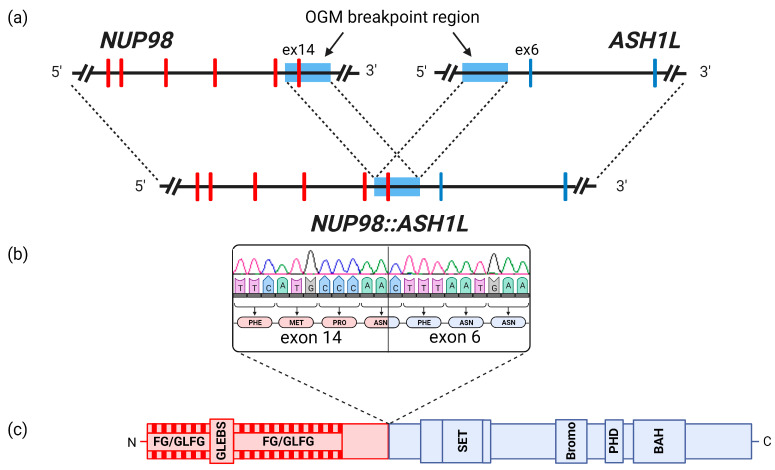
Schematic overview of the *NUP98::ASH1L* fusion. (**a**) Genomic breakpoint (light blue) of the *NUP98::ASH1L* fusion, delimited to an 8 kb region encompassing intron 13 to intron 14 of *NUP98* and intron 5 of *ASH1L* using OGM data. (**b**) RT-PCR analysis showed an in-frame fusion of entire exon 14 of *NUP98* to entire exon 6 of *ASH1L* (sequencing chromatograph exported from SeqMan software). (**c**) Retained protein domains according to the transcript sequence.

The OGM Circos plot at the time of AML diagnosis is depicted in Figure 2c [40]. At this point, OGM revealed complex rearrangements detected as SVs between chromosomes 3, 5, and 10 in VAFs of 21–47% (median 24%), resulting in large losses of genomic material detected as copy number (CN) losses with fractional CNs of approximately 1.0–1.6. Aneuploidy losses of chromosome 16 (CN 1.63) and chromosome 3 (not encompassing the whole chromosome) were detected. Additionally, translocation SVs between chromosomes 1, 11, and 17 in smaller VAFs of 6–10% were present. A generated molecule map (Figure 2b), partially aligned to chromosomes 1 and 11, showed two different SV calls overlapping the genes *NUP98* and *ASH1L*. An inter-chromosomal-translocation SV (VAF 10%) together with an inversion SV (VAF 11%), encompassing an approximately 100 kilobases (kb)-spanning fragment of chromosome 1, was seen. The conjunction of these SVs resulted in a genomic *NUP98::ASH1L* fusion. SVs involving the 3′ part of *NUP98*, leading to a reciprocal *ASH1L::NUP98* fusion, were not detected. The software identified a 11.2 kb-spanning breakpoint region. A DLE-1-targeting site (CTTAAG) was not present in the breakpoint region, according to the chromosome 11 reference (chr11:3.748.481–3.748.486). Therefore, the breakpoint region of the genomic fusion was manually limited to an 8 kb-spanning region located in *NUP98* intron 13–14 and *ASH1L* intron 5 (Figure 2b and Figure 4a). The complete upstream 5′-part of *NUP98* and the complete downstream 3′-part of *ASH1L* were involved in the genomic fusion. The algorithm determining the VAF utilizes the actually detected molecules for each individual SV. This value can differ depending on coverage and labeling, thus possibly resulting in VAF variability.

In the consecutive OGM RVP analysis performed with bone marrow aspirate prior to alloHSCT, rare translocation SVs between chromosomes 3, 5, and 10 were still present in 2–13% VAF (median 9%). Additionally, the translocation and inversion SVs resulting in the *NUP98::ASH1L* fusion remained present in 5% and 6% VAF. No confident CNVs and no aneuploidies were detected.

**Figure 3:**
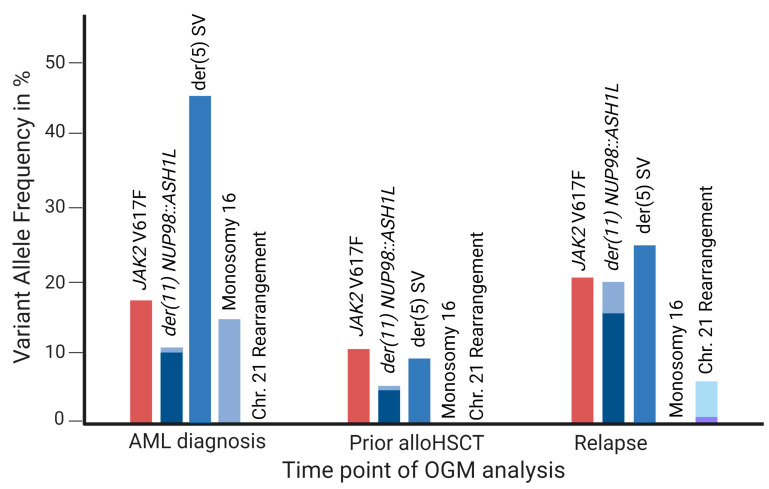
Application of OGM as a genome-wide tool for disease monitoring in AML. VAFs of *JAK2* V617F as detected by qPCR (red columns) and VAFs of selected rare OGM structural and copy number variants (blue columns) relative to the time points of OGM analyses; the discrepancy in output VAFs between translocation and inversion SVs leading to der(11) *NUP98::ASH1L* is displayed in lighter blue.

**Figure A1:**
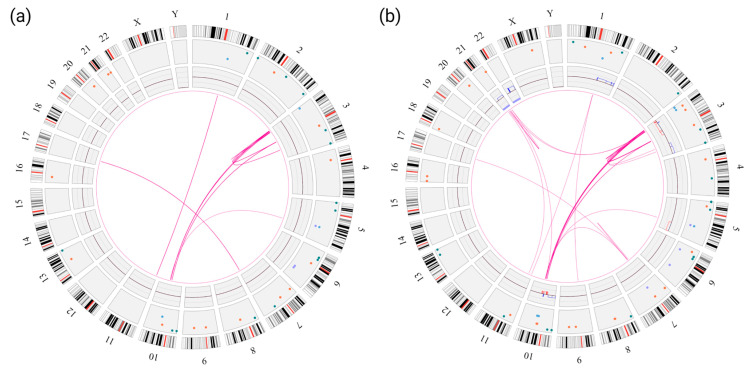
Circos plots of consecutive OGM RVP analyses filtered for confident SVs occurring in ≤1% of controls and confident CNVs; (**a**) prior to alloHSCT (+155 days); (**b**) relapse (+225 days).

At relapse (day +63 after alloHSCT), the *NUP98::ASH1L* fusion was present in 20% VAF, while the translocation SVs between 3, 5, and 10 were present in VAFs of 1–36% (median 15%). The translocation and inversion SVs, resulting in the *NUP98::ASH1L* fusion, were present in 16% and 20% VAF, respectively. While an aneuploidy loss of chromosome 16 was absent at relapse compared to AML diagnosis, additional aberrations, such as translocation SVs involving chromosomes 1, 7, 9, 17, and 21, were present. The aneuploidy CN gains of chromosomes 21 (CN 2.44) and 22 (CN 2.88; likely reflecting tetrasomy 22) were detected. The VAFs of selected OGM variants and the VAF of *JAK2* V617F, as detected by qPCR at all time points of OGM analyses, are depicted in Figure 3 and Table 2. Circos plots of both consecutive OGM analyses are depicted in Appendix B Figure A1.

### 3.3. Confirmation of the NUP98::ASH1L Gene Fusion

To validate the *NUP98::ASH1L* gene fusion in OGM and determine its transcription, RT-PCR and consecutive Sanger sequencing were applied to bone marrow samples isolated at initial AML diagnosis. Here, an in-frame fusion transcript of *NUP98* exon 14 to *ASH1L* exon 6 was identified (Figure 4b).

For *NUP98*, a typical fusion breakpoint downstream of the repeat regions encoding Gly-Leu-Phe-Gly and Phe-Gly (GLFG/FG) amino acids was identified [4,41,42,43]. While the upstream DNA-binding AT-hook motif (exons 2–4) of *ASH1L* was not included in the transcript, the SET-domain (Su(var)3-9, Enhancer-of-zeste, and Trithorax-domain) and its associated AWS-domain (associated with SET) and post-SET-domain were retained. Additionally, the three downstream chromatin reader domains, namely plant homeodomain (PHD)-type zinc finger, bromo domain (BD), and bromo-adjacent homology (BAH) domain, were retained in the transcript (Figure 4c) [44].

## 4. Discussion

### 4.1. OGM as a Tool for Cytogenetic Diagnostics of AML

The exact classification of disease is essential for prognosis and treatment decisions in AML. The WHO recently updated their classification system. For the group of AML with defining genetic abnormalities, it now recognizes—apart from known fusions, e.g., *PML::RARA*, *RUNX1::RUNX1T1* or *CBFB::MYH11*—rearrangements involving *KMT2A*, *MECOM*, and *NUP98*, irrespective of the fusion partner, as disease-defining, even with blast counts below 20% [18]. The novel ELN 2022 recommendations for diagnosis and management of AML in adults now recognize three different AML subtypes defined by *NUP98*-rearrangements, two defined by specific fusions (*::NSD1* and *::KDM5A)* and one defined by *NUP98*-rearrangements other than *NSD1* or *KDM5A* [1].

*NUP98::ASH1L* now represents a novel *NUP98* fusion in this category detected in a case of AML post-MDS/MPN-RS-T. In *NUP98* rearrangements, there is a wide variety of fusion partners. Bertrums et al. recently identified 13 different fusion partners for *NUP98* via transcriptome analysis in a cohort of 2235 children suffering from pediatric AML [45]. In total, at least 39 different fusion partners have been described to date, which can be subdivided into homeodomain (HD)-containing genes (at least 11 partners) and non-HD genes (at least 28 partners) [4,22,45,46,47,48]. Intriguingly, *ASH1L* has not been detected as a fusion partner for *NUP98* so far.

There has been a growing amount of evidence that OGM can be an important diagnostic tool in cytogenetic diagnostics in AML with the potential of not only augmenting but replacing other cytogenetic methods [22,23,24,25,26,27,28]. Recently, we have extensively reviewed the current evidence of OGM as a diagnostic tool in AML, comparing it to standard approaches in cytogenetic diagnostics, most importantly CBA, FISH, and CMA [27]. The present work adds another important feature, namely the detection of classification-relevant but so far unknown gene fusions, as exemplified here through the detection of a *NUP98*::*ASH1L* fusion. Especially for *NUP98* rearrangements, the WHO states that these SVs are often cryptic in CBA [18]. Although standard FISH diagnostics would have been able to detect the *NUP98* break-apart, the novel fusion partner would have remained cryptic with standard cytogenetic diagnostic procedures and standard FISH-panels. This underlines the value of techniques such as OGM. 

### 4.2. NUP98::ASH1L in Context of NUP98-Rearrangements

To the best of our knowledge, this is the first description of a *NUP98*::*ASH1L* fusion. It was found in an AML-MRC. The *NUP98::ASH1L* fusion was represented by a combination of an inversion and translocation SV in OGM. The genomic breakpoint could be narrowed down to an 8 kb region by adapting the OGM results. A limitation of the method was that it was not possible to determine whether exon 14 of *NUP98* was included in the genomic fusion. Using RT-PCR, the transcript showed an inclusion of the entire exon (Figure 4a,b).

NUP98 is a protein cofactor for the nuclear export protein exportin-1 (XPO1) [44]. Although it belongs to the nucleoporin subfamily, oncogenic NUP98 fusion proteins only involve the GLFG/FG-rich repeats encoded by the 5′ end of *NUP98* [4,5]. In *NUP98* fusions, these repeats are known to form nuclear puncta via chemical phase separation, which contribute to the leukemogenesis of *NUP98* fusions [49,50].

The upregulation of *HOX* genes is a common driver of leukemogenesis in AML [51]. Many NUP98 fusion oncoproteins, such as NUP98::NSD1, have been shown to bind to *HOX* gene loci and lead to their overexpression. Several NUP98-fusion proteins, such as ::KDM5A, ::NSD1, and ::HOXA9, are known to co-localize and interact with the KMT2A-complex (syn. MLL-complex), which typically leads to the increased expression of targets, most importantly *HOX* genes. In vivo, the *NUP98::HOXA9* fusion gene needs functional KMT2A for leukemogenesis [49,52,53]. In contrast, the rare *NUP98::KMT2A* fusion seems to unfold its leukemogenic potential through cell cycle alteration rather than through *HOX* gene upregulation [54,55]. Aside from the variable pathogenetic roles of different *NUP98*-fusions, *NUP98*-rearranged murine models show a huge variability in phenotypes such as myeloproliferation, myelodysplasia, and secondary or de novo leukemic transformation [56,57,58,59,60]. Thus, *NUP98*-fusions are far from being understood and—depending on the fusion partner—drive leukemogenesis in different ways from both a pathogenetical and cytomorphological perspective.

As the N-terminal region of *NUP98*, which is typically involved in these rearrangements, seems unlikely to be targetable, independent researchers sought to address the large group of variable C-terminal fusion partners [5,61,62]. A subset of non-HD *NUP98* partners are associated with epigenetic regulation and show common features, such as SET-domains (*ASH1L*, *KMT2A*, *NSD1*, *NSD3*, *SET*, *SETBP1*), PHD-type zinc fingers (*ASH1L*, *BPTF*, *JADE2*, *KDM5A*, *KMT2A*, *NSD1*, *NSD3*, *PHF23*, *TAF3*), or bromo-domains (*ASH1L*, *BPTF*, *BRWD3*, *KMT2A*) [21,45,47,63,64,65,66,67,68,69,70]. These may represent subgroups with distinct biological functions and are potentially directly targetable, as has been shown for the PHD of the NUP98::PHF23 oncoprotein in AML cell lines through disulfiram, a drug currently used for the treatment of chronic alcohol abuse, as it inhibits the PHD-containing acetaldehyde dehydrogenase needed for alcohol metabolism [71,72,73,74]. Other targets might be pathway-related proteins or co-occurring mutations, such as the *NUP98::NSD1*-associated *FLT3*-internal tandem duplication (*FLT3*-ITD), where inhibitors are part of current standard therapies, or, as has been recently shown, interferon signaling as a putative target pathway in *FLT3*-ITD-mutated *NUP98::HOXD13* AML cell lines [1,75,76]. 

In *NUP98*-rearranged neoplasia, the 5′-*NUP98::partner*-3′ genomic fusion must typically be transcribed and translated to an oncoprotein to unfold its leukemogenic potential. Therefore, *NUP98*-rearrangements need to generate a gene fusion open reading frame (GF-ORF). Of all 35 fusion partners mentioned in a review published in 2020 by Michmerhuizen et al., only the rare *NUP98*-fusions *::IQCG*, *::RARG,* and *::MLLT10* had DNA strand orientations that required a more complex genomic rearrangement than a single translocation or inversion, as shown for the novel *NUP98::ASH1L* [4,77,78,79]. Two other exceptionally rare *NUP98*-fusions, namely *::BRWD3* and *::TAF3*, also fell in this category [45,46,47]. The necessity of a more complex rearrangement in these cases with more than two chromosomal breakpoints might partially explain why *NUP98::ASH1L* has not been described before.

### 4.3. Role of ASH1L in Leukemia and Putative Implications for NUP98::ASH1L

*ASH1L* is a gene encoding a histone lysine methyltransferase involved in the epigenetic regulation of chromatin through histone 3 lysine 4 and 36 methylation (H3K4 and H3K36). H3K36 mediates DNA repair and is an important regulator of cell differentiation and growth. ASH1L preferentially methylates H3K36 (H3K36me2) [80,81]. Overexpression, copy number amplification, and mutations of *ASH1L* are recurrently found in different neoplasia, including breast and thyroid cancer [82,83,84,85].

ASH1L is a crucial regulator of the KMT2A-complex, as it enables the recruitment of KMT2A through its cofactor PSIP1 (syn. LEDGF) to multiple target genes associated with leukemic transformation. Via this pathway, ASH1L regulates gene transcription, including homeobox (*HOX*) genes. The histone lysine demethylase KDM2A was described to be an opposing regulator of ASH1L by specifically demethylating H3K36me2, which leads to the dissociation of KMT2A/PS1P1 and the decreased description of KMT2A target leukemia genes. In hematopoietic stem and progenitor cells, *ASH1L* and *KMT2A* are transcribed at high levels and expression decreases with myeloid differentiation. For KDM2A, this relation is inverse [81,82,86,87]. 

The cooperation of KMT2A and ASH1L is required for the effective transcription of *HOX* genes. Only the co-expression of both genes resulted in over 100-fold-increased activation in the promotor regions of *HOX* genes in cell lines. Moreover, the standalone C-terminal part of ASH1L, with deleted N-terminal DNA-binding motifs, is sufficient to induce adequate *HOX* gene expression in cooperation with KMT2A [86,88]. In the *NUP98::ASH1L* fusion described here, the C-terminal part was completely retained, whereas the DNA-binding motifs coded by *ASH1L* exons 2–4 were not present in the fusion. Therefore, the NUP98::ASH1L oncoprotein might be capable of *HOX* gene regulation despite the lack of the ASH1L N-terminus.

ASH1L plays an important role in the pathogenesis of 5′-*KMT2A*-rearranged AML, as has recently been shown [62,82,89]. It is a potential future target in this high-risk subgroup. An inhibitor of the SET-domain of ASH1L showed promising results in *KMT2A*-rearranged in vitro models. The inhibition of ASH1L’s SET-domain led to downregulation of the *HOX* gene expression and induced apoptosis and differentiation in *KMT2A::MLLT3*-transformed leukemia cells [62].

**Figure 5:**
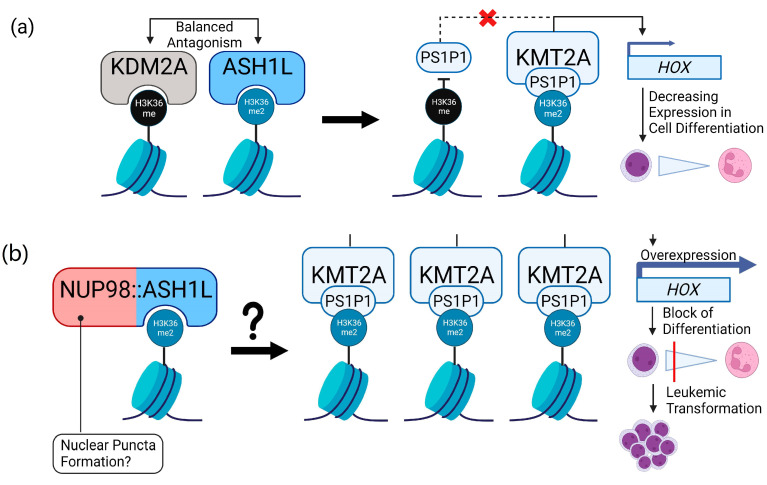
Role of ASH1L in H3K36-regulation and potential implications for NUP98::ASH1L. (**a**) The role of ASH1L in H3K36-dimethylation in leukemia (adaption of [62,80,81,82,87,88,89,90]); KDM2A and ASH1L are functional antagonists leading to demethylation of H3K36me2 and dimethylation of H3K36 respectively; H3K36me2, dimethylated by ASH1L, enables the recruitment of KMT2A via its cofactor PS1P1, leading to the upregulation of *HOX* cluster genes; PS1P1 is not able to bind to H3K36me, that was demethylated by KDM2A, therefore inhibiting the KMT2A-related *HOX* gene expression. (**b**) Putative implications for the NUP98::ASH1L oncoprotein: increased H3K36 methylation might lead to the enhanced recruitment of PS1P1 and KMT2A and, therefore, the upregulation of oncogenes, such as *HOX* genes; it is to be determined whether NUP98::ASH1L mediates its leukemic capabilities via the formation of nuclear puncta as in other related NUP98-fusions [49,50].

Although speculative, we hypothesize that the *NUP98*::*ASH1L* fusion leads to increased H3K36me2, enabling PS1P1 recruitment to ASH1L-written H3K36me2 marks. Therefore, elevated KMT2A/MLL complex epigenetic regulation would lead to the overexpression of KMT2A-regulated oncogenes, such as *HOX* genes (Figure 5). A similar mechanism was previously described in the related fusion *NUP98::NSD1*, where H3K36-methylation and -acetylation through the oncoprotein led to a high level *HOX* expression [56]. Functional experiments are needed to verify this hypothesis and to analyze through which underlying mechanisms the NUP98::ASH1L oncoprotein potentially mediates leukemic transformation. Moreover, the retained SET-domain of NUP98::ASH1L might be addressable in the future, as targeting of the SET domain of wildtype ASHL1 was successful in *KMT2A*-rearranged cell lines.

### 4.4. OGM as a Tool for Disease Monitoring in AML

The case presented in this work represents secondary AML evolving from a *JAK2* mutated MDS/MPN. At the time-point of transformation, the blast count in the bone marrow was between 60% and 70%, whereas the VAF of the known *JAK2* V617F variant and the novel *NUP98::ASH1L* fusion were around 17% and 10%, respectively (Table 1). Although comparing the results of different techniques, this ratio remained roughly the same before alloHSCT. Despite the initial response, there was an early hematological and molecular relapse. 

In the future, OGM might prove to be another helpful tool in disease monitoring in a subgroup of cases. Of course, in most cases, amplification-based methods will continue to show a more sensitive detection of small genetic variants compared to OGM on the Saphyr platform with the current settings (the coverage in our work was, on average, around 300- 600-fold). Nevertheless, once SVs have been detected, no matter whether they are relevant for disease biology or not, they can be followed up using this method. Unlike in acute lymphoblastic leukemia, most centers do not search for a distinct measurable residual disease (MRD) marker in their AML patients, apart from the predefined panels. OGM would need some improvement in coverage to compete with other methods used in this context, such as the combination of panoptical methods, flow cytometry, and FISH. In the case of a lack of small genetic variants for the detection of MRD via amplification-based techniques, OGM might become a helpful tool for the determination of remission for this subgroup of patients in the future. This, naturally, must be proven in a larger context.

## 5. Conclusions

In this work, we exemplarily present OGM as a tool for high-resolution SV disease monitoring and describe *ASH1L* as a novel fusion partner for *NUP98* in a case of secondary AML. We furthermore show the capability of OGM in aiding the generation of a hypothesis for the mechanism of this variant regarding disease biology. This work highlights the value of OGM for enhanced cytogenetic diagnostics and subsequent relevance for classification and stratification by detecting known and unknown fusion partners for genes such as *NUP98*. Moreover, there seems to be a place for genome-wide, high-resolution cytogenetics for monitoring disease, especially if monitoring via amplification-based methods is not possible. Finally, from a scientific point of view, the method potentially helps to identify genomic regions of interest involved in disease evolution. This should be addressed through future research. In conclusion, this work offers possibilities to further evaluate novel genetic findings functionally and to study serial samples over time.

## Figures and Tables

**Table 1:** Summary of cytomorphologic, histopathologic, and genetic results. CBA karyotypes were summarized as metaphases with ≥3 aberrations (complex) and normal karyotypes (46,XX); CR = complete remission; n.a. = not available; Trans. = Translocation; *NUP98*-r = *NUP98*-rearrangement. Myeloproliferative disease was diagnosed approx. 7 years prior to AML with a VAF of *JAK2* V617F of 27% in peripheral blood.

Time Point	Days after Diagnosis (Days after alloHSCT)	Cyto- Morphology	Histology	Chromosomal Banding Analysis ^1^	*JAK2* V617FVAF	Donor Chimerism	*NUP98*-r VAF
AML diagnosis	0	60–70% blasts	n.a.	Complex [29]	17.5%	-	10% Trans., 11% Inversion
Post-Induction I	+35	CR	CR	n.a.	n.a.	-	n.a.
Post-Induction II	+69	CR	CR	46,XX [25]	<0.5%	-	n.a.
Post-Consolidation	+136	CR	5% blasts	n.a.	n.a.	-	n.a.
Prior to alloHSCT	+155 (−7)	CR	15% blasts	n.a.	11.6%	-	5% Trans., 4% Inversion
Post alloHSCT	+182 (+20)	CR	CR	Complex [6/23], 46,XX [17/23]	n.a.	98%	n.a.
Monitoring	+204 (+42)	n.a.	n.a.	n.a.	1.3% ^2^	n.a.	n.a.
Relapse	+225 (+63)	Left shift, single blasts	50% blasts	Complex [25]	21.1%	63%	16% Trans., 20% Inversion
Death	+269 (+107)						

^1^ For detailed karyotypes see Appendix A.1. ^2^ For this analysis, DNA was isolated from peripheral blood instead of bone marrow aspirate.

**Table 2:** Summary of OGM findings used for disease monitoring. VAF values in this table were used to create Figure 3. ChrA and ChrB refer to chromosomes involved in the SV. CN = Copy number.

ChrA	ChrB	ChrA Reference	ChrB Reference	SV Type	Genes	Time Point	VAF
Translocation SV ogm[GRCh37] t(1;11)(q22;p15.4) indicating *NUP98::ASH1L*							
1	11	155384865	3755020	Translocation	*ASH1L*, *NUP98*	AML diagnosis Prior alloHSCT Relapse	10% 5% 16%
Inversion SV ogm[GRCh37] inv(1)(q22q22) indicating *NUP98::ASH1L*							
1	1	155349797	155279255	Inversion	*ASH1L*	AML diagnosis Prior to alloHSCT Relapse	11% 4% 20%
Translocation SV indicating der(5) ogm[GRCh37] t(5;10)(q31.1;q22.2)							
5	10	132131355	75647791	Translocation	-	AML diagnosis Prior to alloHSCT Relapse	46% 9% 25%
Translocation SVs indicating Chr21-Rearrangement (only present at Relapse)							
3	21	16778711	30261569	Translocation	-	Relapse	2%
3	21	18733184	42711338	Translocation	*FAM3B*	Relapse	3%
10	21	72180437	34005936	Translocation	*EIF4EBP2*, *SYNJ1*	Relapse	6%
11	21	21948118	17221321	Translocation	*USP25*	Relapse	3%
21	21	24252871	42525873	Translocation	-	Relapse	1%
21	21	24495432	29856259	Translocation	-	Relapse	3%
**Chr**		**Reference Start**	**Reference End**	**CNV Type**	**fractional CN**	**Time point**	**VAF**
CNV losses indicating Monosomy 16 (only present at AML diagnosis)							
16		2263650	21369108	CNV loss	1.67	AML diagnosis	16%
16		22822268	32019651	CNV loss	1.69	AML diagnosis	16%
16		46438848	85148570	CNV loss	1.68	AML diagnosis	16%

## Data Availability

OGM RVP results can be found in Supplementary Tables S1–S4. Other data obtained and analyzed in this study are available from the corresponding author upon request.

## Supplementary Materials

The following supporting information can be downloaded at: https://www.mdpi.com/article/10.3390/cancers15112942/s1, Table S1: Molecule Quality Parameter after Rare Variant Analysis; Table S2: Rare and Confident Structural Variants; Table S3: Confident Copy Number Variants, Table S4: Confident Aneuploidy Calls.

## Appendix A

### Appendix A.1

Detailed Results of Conventional Karyotyping for Table 1:

- Initial AML diagnosis:
  44~51,XX,-3,add(3)(p11),+add(3)(p11),-10,der(11)t(1;11)(q21;p15),+21,+1~4mar[cp29]
- Post-Induction II:
  46,XX [25]
- Post-alloHSCT:
  46~49,XX,add(3)(p11),-10,der(11)t(1;11)(q21;p15),+21,+22,1~2mar,?inc[cp3]/
  49~50,XX,-3,-10,der(11)t(1:11)(q21;p15),+21,+22,+3~4mar,?inc[cp3]/
  46,XX [17]
- Relapse:
  46~49,XX,-3,-10,der(11)t(1;11)(q21;p15),+21,+22,+1~5mar,?inc[cp14]/
  45~49,XX,add(3)(p11),-10,der(11)t(1;11)(q21;p15),+21,+22,+2~5mar,?inc[cp8]/
  48~49,XX,add(3)(p11),-10,der(11)t(1;11)(q21;p15),add(11)(p1?4),+21,+22,+2~6mar,?inc[cp3]
