# Supplementary material for: Novel NUP98::ASH1L Gene Fusion in Acute Myeloid Leukemia Detected by Optical Genome Mapping
Source: Cancers (Basel). 2023 May 27;15(11):2942. doi: 10.3390/cancers15112942 (PMC10252090; doi:10.3390/cancers15112942)
Supplement: Supplementary file 1 [file cancers-15-02942-s001.zip › cancers-2386029-supplementary.pdf]

**Supplementary Table 1:** Optical Genome Mapping - Molecule Quality Parameter after Rare Variant Analysis

| **OGM Molecule Quality Data** | **AML diagnosis (0d)** | **Prior SCT (+155d)** | **Relapse (+225d)** |
|---|---|---|---|
| Access Version | 1.7 | 1.7 | 1.7 |
| Solve Version | Solve3.7_10192021_74_1 | Solve3.7_10192021_74_1 | Solve3.7_10192021_74_1 |
| RefAligner Version: | 12463 | 12463 | 12463 |
| NLV | 7.176 | 11.275 | 10.315 |
| PLV | 2.745 | 2.61 | 2.732 |
| bpp | 493.72 | 491 | 486.07 |
| res | 2.608 | 2.589 | 2.582 |
| sd | 0.01303 | 0.01901 | 0.01854 |
| sf | 0.09035 | 0.1063 | 0.10524 |
| sr | 0.01696 | 0.01809 | 0.01693 |
| Total number of molecules | 4351058 | 9344283 | 9220623 |
| Total length (Mbp) | 1291978.923 | 2369060.204 | 2343890.409 |
| Average length (kbp) | 296.934 | 253.53 | 254.201 |
| Molecule N50 (kbp) | 313.427 | 257.705 | 257.703 |
| Label density (/100kb) | 16.06 | 15.337 | 15.319 |
| Total number of aligned molecules | 4180719 | 8686723 | 8665191 |
| Fraction of aligned molecules | 0.961 | 0.93 | 0.94 |
| Total molecule align length (Mbp) | 1114275.8 | 1937706.4 | 1929169.7 |
| Total reference align length (Mbp) | 1120158.3 | 1948007.2 | 1938732.3 |
| Effective coverage of reference (X): | 361.846 | 629.267 | 626.271 |
| Average aligned length (kbp) | 266527.3 | 223065.3 | 222634.4 |
| Fraction aligned length | 0.904 | 0.887 | 0.894 |
| Average confidence | 43.7 | 33.6 | 34.5 |
| Deletions | 811 | 857 | 983 |
| Insertions | 825 | 839 | 1026 |
| Duplications | 126 | 111 | 179 |
| Inversion breakpoints | 524 | 464 | 522 |
| Interchr. translocation breakpoints: | 32 | 10 | 32 |
| Intrachr. translocation breakpoints: | 15 | 8 | 19 |

**Supplementary Table 2:** Optical Genome Mapping - Rare Variant Pipeline – Rare and Confident Structural Variants

| Time Point of Analysis (Day) | ChrA | ChrB | Reference Start | Reference End | Confi-dence | Type | Orien-tation | VAF | Size in bases | presence in % of controls | Overlap Genes | Putative Gene Fusion | Self mole-cule count | Result of OGM adapting ISCN |
|---|---|---|---|---|---|---|---|---|---|---|---|---|---|---|
| AML diagnosis (0d) | 2 | 2 | 87083645 | 87097695 | 0.99 | deletion | NA | 0.28 | 4724 | 0 | RMND5A;CD8B;ANAPC1P1 | CD8B-ANAPC1P1 | 58 | ogm[GRCh37] 2p11.2(87083645_87097695)x1 |
| AML diagnosis (0d) | 3 | 3 | 111554983 | 111585361 | 0.99 | deletion | NA | 0.71 | 16189 | 0 | PLCXD2;PHLDB2 | PLCXD2-PHLDB2 | 122 | ogm[GRCh37] 3q13.2(111554983_111585361)x1 |
| AML diagnosis (0d) | 3 | 3 | 6997453 | 9689847 | 0.99 | deletion | NA | 0.4 | 2566875 | 0 | GRM7;AK124857;LMCD1-AS1;U4atac;LMCD1;LINC00312;SSUH2;CAV3;OXTR;Mir_548;RAD18;SRGAP3;BC041457;SRGAP3-AS3;THUMPD3;SETD5-AS1;SETD5;LHFPL4 | - | 78 | ogm[GRCh37] 3p26.1p25.3(6997453_9689847)x1 |
| AML diagnosis (0d) | 3 | 3 | 6997453 | 9689847 | 0.99 | deletion | NA | 0.4 | 2567122 | 0 | GRM7;AK124857;LMCD1-AS1;U4atac;LMCD1;LINC00312;SSUH2;CAV3;OXTR;Mir_548;RAD18;SRGAP3;BC041457;SRGAP3-AS3;THUMPD3;SETD5-AS1;SETD5;LHFPL4 | - | 80 | ogm[GRCh37] 3p26.1p25.3(6997453_9689847)x1 |
| AML diagnosis (0d) | 3 | 3 | 87218114 | 87594795 | 0.99 | deletion | NA | 0.19 | 134919 | 0 | MIR4795;CHMP2B;POU1F1 | - | 29 | ogm[GRCh37] 3p11.2(87218114_87594795)x1 |
| AML diagnosis (0d) | 3 | 3 | 87449156 | 87754519 | 0.97 | deletion | NA | 0.21 | 281369 | 0 | - | - | 56 | ogm[GRCh37] 3p11.2(87449156_87754519)x1 |
| AML diagnosis (0d) | 3 | 3 | 9782366 | 12785919 | 0.99 | deletion | NA | 0.45 | 2962520 | 0 | BRPF1;OGG1;CAMK1;AX748417;TADA3;TTLL3;RPUSD3;CIDEC;JAGN1;IL17RE;IL17RC;CRELD1;PRRT3;PRRT3-AS1;EMC3;AX747493;LOC401052;FW339974;FANCD2;FANCD2OS;BRK1;VHL;IRAK2;TATDN2;LINC00852;GHRLOS;GHRL;SEC13;ATP2B2;MIR885;LINC00606;SLC6A11;SLC6A1;SLC6A1-AS1;HRH1;ATG7;AX748267;VGLL4;TAMM41;DQ583118;SYN2;TIMP4;PPARG;TSEN2;C3orf83;MKRN2;RAF1;TMEM40 | BRPF1-TMEM40 | 90 | ogm[GRCh37] 3p25.3p25.2(9782365_12785919)x1 |
| AML diagnosis (0d) | 4 | 4 | 40566455 | 40581577 | 0.99 | deletion | NA | 0.54 | 7105 | 0.6 | RBM47 | - | 111 | ogm[GRCh37] 4p14(40566455_40581577)x1 |
| AML diagnosis (0d) | 7 | 7 | 62323427 | 62371140 | 0.99 | deletion | NA | 0.7 | 5976 | 0.6 | - | - | 64 | ogm[GRCh37] 7q11.21(62323427_62371140)x1 |
| AML diagnosis (0d) | 7 | 7 | 9901592 | 9914178 | 0.99 | deletion | NA | 0.45 | 5360 | 0 | - | - | 140 | ogm[GRCh37] 7p21.3(9901592_9914178)x1 |

| | | | | | | | | | | | | | | |
|---|---|---|---|---|---|---|---|---|---|---|---|---|---|---|
| AML diagnosis (0d) | 8 | 8 | 70319014 | 70333359 | 0.99 | deletion | NA | 0.54 | 5338 | 0 | - | - | 144 | ogm[GRCh37] 8q13.2(70319014_70333359)x1 |
| AML diagnosis (0d) | 9 | 9 | 128496517 | 128507523 | 0.99 | deletion | NA | 0.71 | 5027 | 0 | - | - | 18 | ogm[GRCh37] 9q33.3(128496517_128507523)x1 |
| AML diagnosis (0d) | 9 | 9 | 128501229 | 128507163 | 0.99 | deletion | NA | 0.71 | 4380 | 0 | - | - | 144 | ogm[GRCh37] 9q33.3(128501229_128507163)x1 |
| AML diagnosis (0d) | 9 | 9 | 65843777 | 65858302 | 0.99 | deletion | NA | 0.41 | 11144 | 0.6 | - | - | 17 | ogm[GRCh37] 9q12(65843777_65858302)x1 |
| AML diagnosis (0d) | 9 | 9 | 95167003 | 95268123 | 0.99 | deletion | NA | 0.51 | 97459 | 0 | CENPP;OMD;ASPN;ECM2 | - | 124 | ogm[GRCh37] 9q22.31(95167003_95268123)x1 |
| AML diagnosis (0d) | 10 | 10 | 63429717 | 66057070 | 0.99 | deletion | NA | 0.41 | 2621700 | 0 | C10orf107;ARID5B;MIR548AV;RTKN2;ZNF365;ADO;EGR2;NRBF2;JMJD1C;AX747628;MIR1296;JMJD1C-AS1;REEP3 | - | 77 | ogm[GRCh37] 10q21.2q21.3(63429717_66057070)x1 |
| AML diagnosis (0d) | 16 | 16 | 23932554 | 23944177 | 0.99 | deletion | NA | 0.64 | 8130 | 0.6 | PRKCB | - | 128 | ogm[GRCh37] 16p12.2(23932554_23944177)x1 |
| AML diagnosis (0d) | 20 | 20 | 44336706 | 44386740 | 0.99 | deletion | NA | 0.49 | 30726 | 0.6 | WFDC13;SPINT4 | - | 112 | ogm[GRCh37] 20q13.12(44336706_44386740)x1 |
| AML diagnosis (0d) | 21 | 21 | 47038229 | 47058698 | 0.99 | deletion | NA | 0.33 | 6529 | 0 | - | - | 152 | ogm[GRCh37] 21q22.3(47038229_47058698)x1 |
| AML diagnosis (0d) | 1 | 1 | 103781820 | 103795767 | 0.99 | insertion | NA | 0.11 | 62347 | 0 | - | - | 18 | ogm[GRCh37] ins(1;?)(p21.1;?) |
| AML diagnosis (0d) | 1 | 1 | 225379891 | 225394366 | 0.99 | insertion | NA | 0.58 | 5969 | 0 | DNAH14 | - | 227 | ogm[GRCh37] ins(1;?)(q42.12;?) |
| AML diagnosis (0d) | 2 | 2 | 234232954 | 234236530 | 0.99 | insertion | NA | 0.67 | 10670 | 0 | SAG | - | 175 | ogm[GRCh37] ins(2;?)(q37.1;?) |
| AML diagnosis (0d) | 3 | 3 | 139131709 | 139137532 | 0.99 | insertion | NA | 0.32 | 6047 | 0.6 | - | - | 110 | ogm[GRCh37] ins(3;?)(q23;?) |
| AML diagnosis (0d) | 5 | 5 | 33059463 | 33064516 | 0.99 | insertion | NA | 0.57 | 6440 | 0 | - | - | 182 | ogm[GRCh37] ins(5;?)(p13.3;?) |
| AML diagnosis (0d) | 6 | 6 | 124456554 | 124456554 | 0.99 | insertion | NA | 0.47 | 38227 | 0 | NKAIN2 | - | 106 | ogm[GRCh37] ins(6;?)(q22.31;?) |
| AML diagnosis (0d) | 6 | 6 | 40586940 | 40591956 | 0.99 | insertion | NA | 0.5 | 6141 | 0 | - | - | 155 | ogm[GRCh37] ins(6;?)(p21.1;?) |
| AML diagnosis (0d) | 7 | 7 | 102314052 | 102331912 | 0.99 | insertion | NA | 0.5 | 96614 | 0 | AK301666 | - | 61 | ogm[GRCh37] ins(7;?)(q22.1;?) |
| AML diagnosis (0d) | 10 | 10 | 1272968 | 1284604 | 0.99 | insertion | NA | 0.52 | 76622 | 0 | ADARB2 | - | 73 | ogm[GRCh37] ins(10;?)(p15.3;?) |

| | | | | | | | | | | | | | | |
|---|---|---|---|---|---|---|---|---|---|---|---|---|---|---|
| AML diagnosis (0d) | 10 | 10 | 18896114 | 18916103 | 0.99 | insertion | NA | 0.47 | 10120 | 0 | NSUN6 | - | 153 | ogm[GRCh37] ins(10;?)(p12.31;?) |
| AML diagnosis (0d) | 10 | 10 | 47051136 | 47067228 | 0.99 | insertion | NA | 0.03 | 10653 | 0 | - | - | 16 | ogm[GRCh37] ins(10;?)(q11.22;?) |
| AML diagnosis (0d) | 11 | 11 | 4310787 | 4349873 | 0.99 | insertion | NA | 0.49 | 96419 | 0.6 | - | - | 76 | ogm[GRCh37] ins(11;?)(p15.4;?) |
| AML diagnosis (0d) | 11 | 11 | 60986963 | 60995766 | 0.99 | insertion | NA | 0.03 | 19188 | 0 | PGA4 | - | 13 | ogm[GRCh37] ins(11;?)(q12.2;?) |
| AML diagnosis (0d) | 12 | 12 | 11541795 | 11550408 | 0.99 | insertion | NA | 0.37 | 79648 | 0 | PRB2 | - | 165 | ogm[GRCh37] ins(12;?)(p13.2;?) |
| AML diagnosis (0d) | 13 | 13 | 113309756 | 113329149 | 0.99 | insertion | NA | 0.54 | 16412 | 0 | C13orf35 | - | 85 | ogm[GRCh37] ins(13;?)(q34;?) |
| AML diagnosis (0d) | 19 | 19 | 53532751 | 53552487 | 0.99 | insertion | NA | 0.81 | 35054 | 0 | ERVV-2 | - | 106 | ogm[GRCh37] ins(19;?)(q13.41;?) |
| AML diagnosis (0d) | 22 | 22 | 21845261 | 21864431 | 0.99 | insertion | NA | 0.08 | 24888 | 0.6 | PI4KAP2 | - | 15 | ogm[GRCh37] ins(22;?)(q11.21;?) |
| AML diagnosis (0d) | 23 | 23 | 61787943 | 61878865 | 0.99 | insertion | NA | 0.2 | 8585 | 0 | - | - | 24 | ogm[GRCh37] ins(23;?)(q11.1;?) |
| AML diagnosis (0d) | 1 | 1 | 155349797 | 155349797 | 0.99 | inversion | NA | 0.11 | -1 | 0 | ASH1L | - | 23 | ogm[GRCh37] inv(1)(q22q22) |
| AML diagnosis (0d) | 1 | -1 | 155279255 | -1 | 0.99 | inversion_partial | NA | 0.11 | -1 | -1 | - | - | -1 | ogm[GRCh37] inv(1)(q22q22) |
| AML diagnosis (0d) | 3 | 3 | 5934692 | 6573492 | 0.98 | inversion | NA | 0.37 | -1 | 0 | AF279782 | - | 97 | ogm[GRCh37] inv(3)(p26.1p26.1) |
| AML diagnosis (0d) | 3 | -1 | 5856855 | -1 | 0.98 | inversion_partial | NA | 0.37 | -1 | -1 | - | - | -1 | ogm[GRCh37] inv(3)(p26.1p26.1) |
| AML diagnosis (0d) | 5 | 5 | 115959912 | 115959912 | 0.74 | inversion | NA | 0.15 | -1 | 0 | - | - | 97 | ogm[GRCh37] inv(5)(q23.1q23.1) |
| AML diagnosis (0d) | 5 | -1 | 116077394 | -1 | 0.74 | inversion_partial | NA | 0.15 | -1 | -1 | - | - | -1 | ogm[GRCh37] inv(5)(q23.1q23.1) |
| AML diagnosis (0d) | 5 | 5 | 115959912 | 115959912 | 1 | inversion | NA | 0.15 | -1 | 0 | - | - | 6 | ogm[GRCh37] inv(5)(q23.1q23.1) |
| AML diagnosis (0d) | 5 | -1 | 116046316 | -1 | 1 | inversion_partial | NA | 0.15 | -1 | -1 | - | - | -1 | ogm[GRCh37] inv(5)(q23.1q23.1) |
| AML diagnosis (0d) | 10 | 10 | 66823948 | 70459974 | 1 | inversion | NA | 0.2 | -1 | 0 | CTNNA3;SnoU40;LRRTM3;HI650153;DNAJC12;U6;SIRT1;HERC4;MYPN;7SK;ATOH7;PBLD;HNRNPH3;RUFY2;DNA2;SLC25A16;TET1 | - | 76 | ogm[GRCh37] inv(10)(q21.3q21.3) |
| AML diagnosis (0d) | 10 | -1 | 70684134 | -1 | 1 | inversion_partial | NA | 0.2 | -1 | -1 | - | - | -1 | ogm[GRCh37] inv(10)(q21.3q21.3) |
| AML diagnosis (0d) | 10 | 10 | 70699027 | 70736620 | 1 | inversion | NA | 0.2 | -1 | 0 | DDX50;DDX21 | - | 66 | ogm[GRCh37] inv(10)(q22.1q22.1) |
| AML diagnosis (0d) | 10 | -1 | 70297175 | -1 | 1 | inversion_partial | NA | 0.2 | -1 | -1 | - | - | -1 | ogm[GRCh37] inv(10)(q22.1q22.1) |

| | | | | | | | | | | | | | | |
|---|---|---|---|---|---|---|---|---|---|---|---|---|---|---|
| AML diagnosis (0d) | 23 | 23 | 148956827 | 148956827 | 0.96 | inversion | NA | 0.13 | -1 | 0 | - | - | 15 | ogm[GRCh37] inv(23)(q28q28) |
| AML diagnosis (0d) | 23 | -1 | 148659537 | -1 | 0.96 | inversion_partial | NA | 0.13 | -1 | -1 | - | - | -1 | ogm[GRCh37] inv(23)(q28q28) |
| AML diagnosis (0d) | 1 | 17 | 154534329 | 1280603 | 0.96 | translocation_interchr | +/- | 0.06 | -1 | 0 | YWHAE | - | 11 | ogm[GRCh37] t(1;17)(q21.3;p13.3) |
| AML diagnosis (0d) | 1 | 11 | 155384865 | 3755020 | 0.87 | translocation_interchr | +/+ | 0.1 | -1 | 0 | ASH1L;NUP98 | ASH1L-NUP98 | 26 | ogm[GRCh37] t(1;11)(q22;p15.4) |
| AML diagnosis (0d) | 3 | 10 | 12842453 | 17364994 | 0.26 | translocation_interchr | +/+ | 0.41 | -1 | 0 | CAND2;ST8SIA6 | CAND2-ST8SIA6 | 92 | ogm[GRCh37] t(3;10)(p25.2;p12.33) |
| AML diagnosis (0d) | 3 | 10 | 16344791 | 72152951 | 1 | translocation_interchr | +/+ | 0.45 | -1 | 0 | OXNAD1 | - | 117 | ogm[GRCh37] t(3;10)(p25.1;q22.1) |
| AML diagnosis (0d) | 3 | 10 | 170076915 | 18677477 | 1 | translocation_interchr | +/+ | 0.23 | -1 | 0 | CACNB2 | - | 63 | ogm[GRCh37] t(3;10)(q26.2;p12.32) |
| AML diagnosis (0d) | 3 | 10 | 8383755 | 71718153 | 1 | translocation_interchr | +/+ | 0.22 | -1 | 0 | LMCD1-AS1;COL13A1 | LMCD1-AS1-COL13A1 | 40 | ogm[GRCh37] t(3;10)(p26.1;q22.1) |
| AML diagnosis (0d) | 3 | 10 | 86634401 | 63706716 | 1 | translocation_interchr | +/+ | 0.44 | -1 | 0 | ARID5B | - | 116 | ogm[GRCh37] t(3;10)(p12.1;q21.2) |
| AML diagnosis (0d) | 3 | 10 | 87218114 | 19452703 | 0.96 | translocation_interchr | +/- | 0.21 | -1 | 0 | - | - | 38 | ogm[GRCh37] t(3;10)(p11.2;p12.31) |
| AML diagnosis (0d) | 3 | 10 | 87593525 | 42603355 | 0.19 | translocation_interchr | +/+ | 0.22 | -1 | 0 | - | - | 48 | ogm[GRCh37] t(3;10)(p11.2;q11.21) |
| AML diagnosis (0d) | 3 | 10 | 87594795 | 19216957 | 1 | translocation_interchr | -/+ | 0.21 | -1 | 0 | - | - | 47 | ogm[GRCh37] t(3;10)(p11.2;p12.31) |
| AML diagnosis (0d) | 3 | 10 | 9689847 | 72541265 | 0.99 | translocation_interchr | +/+ | 0.17 | -1 | 0 | TBATA | - | 36 | ogm[GRCh37] t(3;10)(p25.3;q22.1) |
| AML diagnosis (0d) | 3 | 10 | 9811091 | 17362391 | 0.89 | translocation_interchr | +/+ | 0.24 | -1 | 0 | CAMK1 | - | 27 | ogm[GRCh37] t(3;10)(p25.3;p12.33) |
| AML diagnosis (0d) | 3 | 10 | 9811823 | 17362391 | 0.94 | translocation_interchr | +/+ | 0.24 | -1 | 0 | - | - | 56 | ogm[GRCh37] t(3;10)(p25.3;p12.33) |
| AML diagnosis (0d) | 3 | 10 | 9821683 | 17362391 | 0.72 | translocation_interchr | +/+ | 0.24 | -1 | 0 | TADA3 | - | 10 | ogm[GRCh37] t(3;10)(p25.3;p12.33) |
| AML diagnosis (0d) | 3 | 10 | 9947565 | 62436894 | 0.86 | translocation_interchr | +/+ | 0.21 | -1 | 0 | IL17RE | - | 41 | ogm[GRCh37] t(3;10)(p25.3;q21.2) |
| AML diagnosis (0d) | 3 | 10 | 9954410 | 62436894 | 0.92 | translocation_interchr | +/+ | 0.21 | -1 | 0 | IL17RE | - | 40 | ogm[GRCh37] t(3;10)(p25.3;q21.2) |
| AML diagnosis (0d) | 5 | 10 | 132131355 | 75647791 | 1 | translocation_interchr | +/+ | 0.46 | -1 | 0 | - | - | 97 | ogm[GRCh37] t(5;10)(q31.1;q22.2) |
| AML diagnosis (0d) | 5 | 17 | 167564781 | 77536514 | 0.22 | translocation_interchr | +/- | 0.79 | -1 | 0 | TENM2 | - | 189 | ogm[GRCh37] t(5;17)(q34;q25.3) |
| AML diagnosis (0d) | 7 | 14 | 61676393 | 87129869 | 0.07 | translocation_interchr | +/- | 0.39 | -1 | 0 | - | - | 103 | ogm[GRCh37] t(7;14)(q11.1;q31.3) |

| | | | | | | | | | | | | | |
|---|---|---|---|---|---|---|---|---|---|---|---|---|---|
| AML diagnosis (0d) | 11 | 17 | 111925791 | 16219538 | 0.99 | translocation_interchr | -/+ | 0.09 | -1 | 0 | DLAT;PIGL | DLAT-PIGL | 23 | ogm[GRCh37] t(11;17)(q23.1;p11.2) |
| AML diagnosis (0d) | 1 | 1 | 150764470 | 203733734 | 0.94 | translocation_intrachr | +/+ | 0.09 | -1 | 0 | - | - | 17 | ogm[GRCh37] fus(1;1)(q21.3;q32.1) |
| AML diagnosis (0d) | 3 | 3 | 19043437 | 85941272 | 1 | translocation_intrachr | +/+ | 0.47 | -1 | 0 | CADM2 | - | 126 | ogm[GRCh37] fus(3;3)(p24.3;p12.1) |
| AML diagnosis (0d) | 3 | 3 | 5934692 | 87094899 | 0.52 | translocation_intrachr | +/+ | 0.31 | -1 | 0 | - | - | 86 | ogm[GRCh37] fus(3;3)(p26.1;p12.1) |
| AML diagnosis (0d) | 10 | 10 | 17362391 | 62436894 | 0.95 | translocation_intrachr | +/+ | 0.22 | -1 | 0 | - | - | 41 | ogm[GRCh37] fus(10;10)(p12.33;q21.2) |
| AML diagnosis (0d) | 6 | 6 | 115448373 | 115526974 | -1 | duplication | NA | 0.36 | 78602 | 0 | - | - | 89 | ogm[GRCh37] dup(6)(q22.1q22.1) |
| AML diagnosis (0d) | 6 | 6 | 115449485 | 115556170 | -1 | duplication | NA | 0.36 | 106686 | 0 | - | - | 64 | ogm[GRCh37] dup(6)(q22.1q22.1) |
| AML diagnosis (0d) | 7 | 7 | 143271389 | 143329948 | -1 | duplication | NA | 0.34 | 58560 | 0.6 | CTAGE15;DQ786304;FAM115C | CTAGE15-FAM115C | 61 | ogm[GRCh37] dup(7)(q35q35) |
| AML diagnosis (0d) | 5 | 5 | 115956150 | 116046316 | -1 | duplication_inverted | NA | 0.52 | 90167 | 0 | Mir_633 | - | 43 | ogm[GRCh37] dup(5)(q23.1q23.1) |
| AML diagnosis (0d) | 5 | 5 | 115956150 | 116056399 | -1 | duplication_inverted | NA | 0.52 | 100250 | 0 | Mir_633 | - | 44 | ogm[GRCh37] dup(5)(q23.1q23.1) |
| AML diagnosis (0d) | 11 | 11 | 4143061 | 4331668 | -1 | duplication_inverted | NA | 0.15 | 188608 | 0 | RRM1;LOC100506082 | - | 46 | ogm[GRCh37] dup(11)(p15.4p15.4) |
| AML diagnosis (0d) | 23 | 23 | 148578211 | 148659537 | -1 | duplication_inverted | NA | 0.12 | 81327 | 0 | IDS;IDS2;LINC00893;CXorf40A | - | 42 | ogm[GRCh37] dup(23)(q28q28) |
| AML diagnosis (0d) | 23 | 23 | 148679644 | 148737119 | -1 | duplication_inverted | NA | 0.13 | 57476 | 0 | TMEM185A | - | 15 | ogm[GRCh37] dup(23)(q28q28) |
| AML diagnosis (0d) | 3 | 3 | 87449930 | 88537466 | -1 | duplication_split | NA | 1 | 1087537 | 0 | U6atac;HTR1F;CGGBP1;ZNF654;C3orf38 | - | 34 | ogm[GRCh37] dup(3)(p11.2p11.1) |
| AML diagnosis (0d) | 7 | 7 | 61533417 | 61902991 | -1 | duplication_split | NA | 0.99 | 369575 | 0 | - | - | 6 | ogm[GRCh37] dup(7)(q11.1q11.21) |
| Prior alloHSCT (+155d) | 2 | 2 | 87083127 | 87097695 | 0.99 | deletion | NA | 0.74 | 4925 | 0 | RMND5A;CD8B;ANAPC1P1 | CD8B-ANAPC1P1 | 207 | ogm[GRCh37] 2p11.2(87083127_87097695)x1 |
| Prior alloHSCT (+155d) | 2 | 2 | 87083566 | 87097774 | 0.99 | deletion | NA | 0.74 | 4934 | 0 | RMND5A;CD8B;ANAPC1P1 | CD8B-ANAPC1P1 | 134 | ogm[GRCh37] 2p11.2(87083565_87097774)x1 |
| Prior alloHSCT (+155d) | 2 | 2 | 87083645 | 87097695 | 0.99 | deletion | NA | 0.74 | 4531 | 0 | RMND5A;CD8B;ANAPC1P1 | CD8B-ANAPC1P1 | 128 | ogm[GRCh37] 2p11.2(87083645_87097695)x1 |
| Prior alloHSCT (+155d) | 3 | 3 | 111554983 | 111585361 | 0.99 | deletion | NA | 0.56 | 16119 | 0 | PLCXD2;PHLDB2 | PLCXD2-PHLDB2 | 280 | ogm[GRCh37] 3q13.2(111554983_111585361)x1 |

| | | | | | | | | | | | | | | |
|---|---|---|---|---|---|---|---|---|---|---|---|---|---|---|
| Prior alloHSCT (+155d) | 4 | 4 | 40566455 | 40581577 | 0.99 | deletion | NA | 0.51 | 7078 | 0.6 | RBM47 | - | 282 | ogm[GRCh37] 4p14(40566455_40581577)x1 |
| Prior alloHSCT (+155d) | 6 | 6 | 66238764 | 66266373 | 0.99 | deletion | NA | 0.89 | 2618 | 0 | EYS | - | 55 | ogm[GRCh37] 6q12(66238764_66266373)x1 |
| Prior alloHSCT (+155d) | 7 | 7 | 62323427 | 62371140 | 0.99 | deletion | NA | 0.62 | 6054 | 0 | - | - | 64 | ogm[GRCh37] 7q11.21(62323427_62371140)x1 |
| Prior alloHSCT (+155d) | 7 | 7 | 9901589 | 9914181 | 0.99 | deletion | NA | 0.52 | 5384 | 0 | - | - | 142 | ogm[GRCh37] 7p21.3(9901588_9914181)x1 |
| Prior alloHSCT (+155d) | 7 | 7 | 9901592 | 9914178 | 0.99 | deletion | NA | 0.52 | 5344 | 0 | - | - | 255 | ogm[GRCh37] 7p21.3(9901592_9914178)x1 |
| Prior alloHSCT (+155d) | 8 | 8 | 70319014 | 70333359 | 0.99 | deletion | NA | 0.48 | 5324 | 0.6 | - | - | 257 | ogm[GRCh37] 8q13.2(70319014_70333359)x1 |
| Prior alloHSCT (+155d) | 9 | 9 | 46436460 | 46446136 | 0.99 | deletion | NA | 0.99 | 6079 | 0 | - | - | 91 | ogm[GRCh37] 9p11.2(46436460_46446136)x1 |
| Prior alloHSCT (+155d) | 9 | 9 | 95167003 | 95268123 | 0.99 | deletion | NA | 0.5 | 97463 | 0 | CENPP;OMD;ASPN;ECM2 | - | 184 | ogm[GRCh37] 9q22.31(95167003_95268123)x1 |
| Prior alloHSCT (+155d) | 10 | 10 | 63429717 | 66057070 | 0.99 | deletion | NA | 0.1 | 2621676 | 0 | C10orf107;ARID5B;MIR548AV;RTKN2;ZNF365;ADO;EGR2;NRBF2;JMJD1C;AX747628;MIR1296;JMJD1C-AS1;REEP3 | - | 37 | ogm[GRCh37] 10q21.2q21.3(63429717_66057070)x1 |
| Prior alloHSCT (+155d) | 13 | 13 | 65341820 | 65357226 | 0.99 | deletion | NA | 0.47 | 3756 | 0 | - | - | 256 | ogm[GRCh37] 13q21.31(65341820_65357226)x1 |
| Prior alloHSCT (+155d) | 16 | 16 | 23931090 | 23940422 | 0.99 | deletion | NA | 0.48 | 7787 | 0.6 | PRKCB | - | 196 | ogm[GRCh37] 16p12.2(23931090_23940422)x1 |
| Prior alloHSCT (+155d) | 16 | 16 | 23932554 | 23944177 | 0.99 | deletion | NA | 0.48 | 8121 | 0.6 | PRKCB | - | 205 | ogm[GRCh37] 16p12.2(23932554_23944177)x1 |
| Prior alloHSCT (+155d) | 20 | 20 | 44203215 | 44209690 | 0.99 | deletion | NA | 0.48 | 3180 | 0 | WFDC8 | - | 286 | ogm[GRCh37] 20q13.12(44203215_44209690)x1 |
| Prior alloHSCT (+155d) | 20 | 20 | 44336706 | 44386740 | 0.99 | deletion | NA | 0.51 | 30686 | 0.6 | WFDC13;SPINT4 | - | 175 | ogm[GRCh37] 20q13.12(44336706_44386740)x1 |
| Prior alloHSCT (+155d) | 21 | 21 | 34403641 | 36841365 | 0.99 | deletion | NA | 0.01 | 2420410 | 0 | OLIG1;C21orf54;IFNAR2;IL10RB-AS1;IFNAR1;IFNGR2;TMEM50B;TMEM50B;DNAJC28;GART;SON;DONSON;CRYZL1;ITSN1;intersectin 1 long form;LINC00649;SLC5A3;MRPS6;LINC00310;KCNE2; | - | 5 | ogm[GRCh37] 21q22.11q22.12(34403641_36841365)x1 |

| | | | | | | | | | | | | | | |
|---|---|---|---|---|---|---|---|---|---|---|---|---|---|---|
| | | | | | | | | | | | SMIM11;BC049386;KCNE1;RCAN1;Mir_562;CLIC6;LINC00160;LOC100506385;RUNX1;AX746823;AX813477;RUNX1-IT1;LOC100506403 | | | |
| Prior alloHSCT (+155d) | 21 | 21 | 47038229 | 47058698 | 0.99 | deletion | NA | 0.62 | 6472 | 0 | - | - | 232 | ogm[GRCh37] 21q22.3(47038229_47058698)x1 |
| Prior alloHSCT (+155d) | 1 | 1 | 225388244 | 225394366 | 0.99 | insertion | NA | 0.48 | 6105 | 0 | DNAH14 | - | 349 | ogm[GRCh37] ins(1;?)(q42.12;?) |
| Prior alloHSCT (+155d) | 2 | 2 | 136581892 | 136613712 | 0.99 | insertion | NA | 0.31 | 4539 | 0.6 | LCT;MCM6 | - | 106 | ogm[GRCh37] ins(2;?)(q21.3;?) |
| Prior alloHSCT (+155d) | 2 | 2 | 234232954 | 234236530 | 0.99 | insertion | NA | 0.63 | 10696 | 0 | SAG | - | 259 | ogm[GRCh37] ins(2;?)(q37.1;?) |
| Prior alloHSCT (+155d) | 2 | 2 | 298743 | 310577 | 0.99 | insertion | NA | 0.62 | 3613 | 0.6 | - | - | 327 | ogm[GRCh37] ins(2;?)(p25.3;?) |
| Prior alloHSCT (+155d) | 3 | 3 | 139131709 | 139137532 | 0.99 | insertion | NA | 0.49 | 6084 | 0.6 | - | - | 321 | ogm[GRCh37] ins(3;?)(q23;?) |
| Prior alloHSCT (+155d) | 3 | 3 | 195820356 | 195840292 | 0.99 | insertion | NA | 0.46 | 4780 | 0.6 | - | - | 49 | ogm[GRCh37] ins(3;?)(q29;?) |
| Prior alloHSCT (+155d) | 3 | 3 | 195820356 | 195840886 | 0.99 | insertion | NA | 0.46 | 4098 | 0.6 | - | - | 190 | ogm[GRCh37] ins(3;?)(q29;?) |
| Prior alloHSCT (+155d) | 5 | 5 | 1837315 | 1872381 | 0.99 | insertion | NA | 0.61 | 4926 | 0.6 | - | - | 209 | ogm[GRCh37] ins(5;?)(p15.33;?) |
| Prior alloHSCT (+155d) | 5 | 5 | 33059463 | 33064516 | 0.99 | insertion | NA | 0.51 | 6508 | 0 | - | - | 342 | ogm[GRCh37] ins(5;?)(p13.3;?) |
| Prior alloHSCT (+155d) | 6 | 6 | 30435443 | 30440786 | 0.99 | insertion | NA | 0.48 | 4922 | 0 | - | - | 239 | ogm[GRCh37] ins(6;?)(p21.33;?) |
| Prior alloHSCT (+155d) | 6 | 6 | 31338429 | 31358385 | 0.99 | insertion | NA | 0.56 | 98043 | 0 | - | - | 103 | ogm[GRCh37] ins(6;?)(p21.33;?) |
| Prior alloHSCT (+155d) | 6 | 6 | 40586940 | 40591956 | 0.99 | insertion | NA | 0.49 | 6145 | 0 | - | - | 281 | ogm[GRCh37] ins(6;?)(p21.1;?) |
| Prior alloHSCT (+155d) | 7 | 7 | 102314052 | 102331912 | 0.99 | insertion | NA | 0.24 | 3815 | 0 | AK301666 | - | 77 | ogm[GRCh37] ins(7;?)(q22.1;?) |
| Prior alloHSCT (+155d) | 8 | 8 | 12215199 | 12245552 | 0.99 | insertion | NA | 0.2 | 4043 | 0 | ZNF705A;LOC649352 | - | 81 | ogm[GRCh37] ins(8;?)(p23.1;?) |
| Prior alloHSCT (+155d) | 10 | 10 | 1272968 | 1284604 | 0.99 | insertion | NA | 0.51 | 76648 | 0 | ADARB2 | - | 89 | ogm[GRCh37] ins(10;?)(p15.3;?) |
| Prior alloHSCT (+155d) | 10 | 10 | 18896114 | 18916103 | 0.99 | insertion | NA | 0.49 | 10096 | 0 | NSUN6 | - | 279 | ogm[GRCh37] ins(10;?)(p12.31;?) |
| Prior alloHSCT (+155d) | 13 | 13 | 113309756 | 113329149 | 0.99 | insertion | NA | 0.45 | 16496 | 0 | C13orf35 | - | 174 | ogm[GRCh37] ins(13;?)(q34;?) |
| Prior alloHSCT (+155d) | 1 | 1 | 155349797 | 155349797 | 1 | inversion | NA | 0.04 | -1 | 0.6 | ASH1L | - | 14 | ogm[GRCh37] inv(1)(q22q22) |

| | | | | | | | | | | | | | | |
|---|---|---|---|---|---|---|---|---|---|---|---|---|---|---|
| Prior alloHSCT (+155d) | 1 | -1 | 155279255 | -1 | 1 | inversion_partial | NA | 0.04 | -1 | -1 | - | - | -1 | ogm[GRCh37] inv(1)(q22q22) |
| Prior alloHSCT (+155d) | 3 | 3 | 5935872 | 6573492 | 0.99 | inversion | NA | 0.04 | -1 | 0 | AF279782 | - | 10 | ogm[GRCh37] inv(3)(p26.1p26.1) |
| Prior alloHSCT (+155d) | 3 | -1 | 5856855 | -1 | 0.99 | inversion_partial | NA | 0.04 | -1 | -1 | - | - | -1 | ogm[GRCh37] inv(3)(p26.1p26.1) |
| Prior alloHSCT (+155d) | 5 | 5 | 115974899 | 115974899 | 0.9 | inversion | NA | 0.15 | -1 | 0 | - | - | 27 | ogm[GRCh37] inv(5)(q23.1q23.1) |
| Prior alloHSCT (+155d) | 5 | -1 | 116062655 | -1 | 0.9 | inversion_partial | NA | 0.15 | -1 | -1 | - | - | -1 | ogm[GRCh37] inv(5)(q23.1q23.1) |
| Prior alloHSCT (+155d) | 10 | 10 | 66823948 | 70478435 | 1 | inversion | NA | 0.08 | -1 | 0 | CTNNA3;SnoU40;LRRTM3;HI650153;DNAJC12;U6;SIRT1;HERC4;MYPN;7SK;ATOH7;PBLD;HNRNPH3;RUFY2;DNA2;SLC25A16;TET1 | - | 26 | ogm[GRCh37] inv(10)(q21.3q21.3) |
| Prior alloHSCT (+155d) | 10 | -1 | 70700810 | -1 | 1 | inversion_partial | NA | 0.08 | -1 | -1 | - | - | -1 | ogm[GRCh37] inv(10)(q21.3q21.3) |
| Prior alloHSCT (+155d) | 10 | 10 | 70544201 | 70736620 | 1 | inversion | NA | 0.17 | -1 | 0 | CCAR1;STOX1;DDX50;DDX21 | - | 24 | ogm[GRCh37] inv(10)(q21.3q22.1) |
| Prior alloHSCT (+155d) | 10 | -1 | 70297175 | -1 | 1 | inversion_partial | NA | 0.17 | -1 | -1 | - | - | -1 | ogm[GRCh37] inv(10)(q21.3q22.1) |
| Prior alloHSCT (+155d) | 1 | 11 | 155384865 | 3755020 | 0.69 | translocation_interchr | +/+ | 0.05 | -1 | 0 | ASH1L;NUP98 | ASH1L-NUP98 | 20 | ogm[GRCh37] t(1;11)(q22;p15.4) |
| Prior alloHSCT (+155d) | 3 | 10 | 16344791 | 72152951 | 0.99 | translocation_interchr | +/+ | 0.13 | -1 | 0 | OXNAD1 | - | 23 | ogm[GRCh37] t(3;10)(p25.1;q22.1) |
| Prior alloHSCT (+155d) | 3 | 10 | 8383755 | 71718153 | 0.97 | translocation_interchr | +/+ | 0.08 | -1 | 0 | LMCD1-AS1;COL13A1 | LMCD1-AS1-COL13A1 | 26 | ogm[GRCh37] t(3;10)(p26.1;q22.1) |
| Prior alloHSCT (+155d) | 3 | 10 | 86013858 | 63895519 | 0.6 | translocation_interchr | +/+ | 0.01 | -1 | 0 | CADM2 | - | 6 | ogm[GRCh37] t(3;10)(p12.1;q21.2) |
| Prior alloHSCT (+155d) | 3 | 10 | 86634401 | 63706716 | 0.99 | translocation_interchr | +/+ | 0.08 | -1 | 0 | ARID5B | - | 20 | ogm[GRCh37] t(3;10)(p12.1;q21.2) |
| Prior alloHSCT (+155d) | 5 | 10 | 132131355 | 75647791 | 0.98 | translocation_interchr | +/+ | 0.09 | -1 | 0 | - | - | 23 | ogm[GRCh37] t(5;10)(q31.1;q22.2) |
| Prior alloHSCT (+155d) | 7 | 17 | 154456988 | 27326038 | 0.2 | translocation_interchr | +/+ | 0.26 | -1 | 0 | DPP6;SEZ6 | DPP6-SEZ6 | 168 | ogm[GRCh37] t(7;17)(q36.2;q11.2) |
| Prior alloHSCT (+155d) | 3 | 3 | 5935872 | 87094899 | 0.5 | translocation_intrachr | +/+ | 0.04 | -1 | 0 | - | - | 13 | ogm[GRCh37] fus(3;3)(p26.1;p12.1) |
| Prior alloHSCT (+155d) | 3 | 3 | 7921134 | 15366443 | 0.92 | translocation_intrachr | -/+ | 0.02 | -1 | 0 | SH3BP5 | - | 7 | ogm[GRCh37] fus(3;3)(p26.1;p25.1) |
| Prior alloHSCT (+155d) | 3 | 3 | 11711969 | 141424983 | 0.15 | translocation_intrachr | -/+ | 0.02 | -1 | 0 | - | - | 6 | ogm[GRCh37] fus(3;3)(p25.3;q23) |
| Prior alloHSCT (+155d) | 3 | 3 | 19043437 | 85941272 | 0.99 | translocation_intrachr | +/+ | 0.09 | -1 | 0 | CADM2 | - | 30 | ogm[GRCh37] fus(3;3)(p24.3;p12.1) |

| | | | | | | | | | | | | | | |
|---|---|---|---|---|---|---|---|---|---|---|---|---|---|---|
| Prior alloHSCT (+155d) | 6 | 6 | 115447157 | 115556170 | -1 | duplication | NA | 0.06 | 109014 | 0 | - | - | 19 | ogm[GRCh37] dup(6)(q22.1q22.1) |
| Prior alloHSCT (+155d) | 6 | 6 | 124433875 | 124456554 | -1 | duplication | NA | 0.46 | 22680 | 0 | NKAIN2 | - | 61 | ogm[GRCh37] dup(6)(q22.31q22.31) |
| Prior alloHSCT (+155d) | 5 | 5 | 115956150 | 116056399 | -1 | duplication_inverted | NA | 0.49 | 100250 | 0 | Mir_633 | - | 86 | ogm[GRCh37] dup(5)(q23.1q23.1) |
| Relapse (+225d) | 1 | 1 | 182773347 | 182793139 | 0.99 | deletion | NA | 0.19 | 5368 | 0.6 | NPL | - | 242 | ogm[GRCh37] 1q25.3(182773347_182793139)x1 |
| Relapse (+225d) | 1 | 1 | 73007503 | 73093313 | 0.99 | deletion | NA | 0.23 | 82366 | 0 | - | - | 77 | ogm[GRCh37] 1p31.1(73007503_73093313)x1 |
| Relapse (+225d) | 3 | 3 | 111554983 | 111585361 | 0.99 | deletion | NA | 0.3 | 16165 | 0 | PLCXD2;PHLDB2 | PLCXD2-PHLDB2 | 218 | ogm[GRCh37] 3q13.2(111554983_111585361)x1 |
| Relapse (+225d) | 3 | 3 | 18296458 | 18515116 | 0.97 | deletion | NA | 0.01 | 211886 | 0 | LOC339862;SATB1 | - | 6 | ogm[GRCh37] 3p24.3(18296457_18515116)x1 |
| Relapse (+225d) | 3 | 3 | 39760310 | 39781339 | 0.99 | deletion | NA | 0.36 | 17617 | 0 | - | - | 216 | ogm[GRCh37] 3p22.1(39760310_39781339)x1 |
| Relapse (+225d) | 4 | 4 | 40566455 | 40581577 | 0.99 | deletion | NA | 0.29 | 7104 | 0.6 | RBM47 | - | 202 | ogm[GRCh37] 4p14(40566455_40581577)x1 |
| Relapse (+225d) | 5 | 5 | 21603512 | 21620550 | 0.99 | deletion | NA | 0.19 | 3283 | 0.6 | BC038535 | - | 68 | ogm[GRCh37] 5p14.3(21603511_21620549)x1 |
| Relapse (+225d) | 6 | 6 | 16563958 | 16575942 | 0.99 | deletion | NA | 0.29 | 5628 | 0 | ATXN1 | - | 186 | ogm[GRCh37] 6p22.3(16563958_16575941)x1 |
| Relapse (+225d) | 7 | 7 | 62323427 | 62371140 | 0.99 | deletion | NA | 0.4 | 6008 | 0 | - | - | 45 | ogm[GRCh37] 7q11.21(62323427_62371140)x1 |
| Relapse (+225d) | 7 | 7 | 8881992 | 8897787 | 0.99 | deletion | NA | 0.27 | 6964 | 0 | - | - | 166 | ogm[GRCh37] 7p21.3(8881992_8897787)x1 |
| Relapse (+225d) | 7 | 7 | 9901592 | 9914178 | 0.99 | deletion | NA | 0.25 | 5409 | 0 | - | - | 161 | ogm[GRCh37] 7p21.3(9901592_9914178)x1 |
| Relapse (+225d) | 8 | 8 | 70319014 | 70333359 | 0.99 | deletion | NA | 0.24 | 5348 | 0.6 | - | - | 289 | ogm[GRCh37] 8q13.2(70319014_70333359)x1 |
| Relapse (+225d) | 8 | 8 | 72213283 | 72218721 | 0.99 | deletion | NA | 0.43 | 3084 | 0 | EYA1 | - | 243 | ogm[GRCh37] 8q13.3(72213283_72218721)x1 |

| | | | | | | | | | | | | | | |
|---|---|---|---|---|---|---|---|---|---|---|---|---|---|---|
| Relapse (+225d) | 9 | 9 | 67000179 | 67044327 | 0.99 | deletion | NA | 0.15 | 27700 | 0.6 | LOC286297 | - | 12 | ogm[GRCh37] 9q13(67000178_67044327)x1 |
| Relapse (+225d) | 9 | 9 | 95167003 | 95268123 | 0.99 | deletion | NA | 0.22 | 97409 | 0 | CENPP;OMD;ASPN;ECM2 | - | 90 | ogm[GRCh37] 9q22.31(95167003_95268123)x1 |
| Relapse (+225d) | 10 | 10 | 63429717 | 66057070 | 0.99 | deletion | NA | 0.37 | 2621687 | 0 | C10orf107;ARID5B;MIR548AV;RTKN2;ZNF365;ADO;EGR2;NRBF2;JMJD1C;AX747628;MIR1296;JMJD1C-AS1;REEP3 | - | 203 | ogm[GRCh37] 10q21.2q21.3(63429717_66057070)x1 |
| Relapse (+225d) | 10 | 10 | 65959767 | 66724105 | 0.99 | deletion | NA | 0.01 | 656533 | 0 | ANXA2P3;DJ439558;DJ439576;DJ439561 | - | 5 | ogm[GRCh37] 10q21.3(65959767_66724105)x1 |
| Relapse (+225d) | 11 | 11 | 21834510 | 21955750 | 0.99 | deletion | NA | 0.06 | 114015 | 0 | - | - | 7 | ogm[GRCh37] 11p14.3(21834510_21955750)x1 |
| Relapse (+225d) | 16 | 16 | 23932554 | 23944177 | 0.99 | deletion | NA | 0.25 | 8141 | 0.6 | PRKCB | - | 166 | ogm[GRCh37] 16p12.2(23932554_23944177)x1 |
| Relapse (+225d) | 16 | 16 | 23932554 | 23944177 | 0.99 | deletion | NA | 0.25 | 8150 | 0.6 | PRKCB | - | 158 | ogm[GRCh37] 16p12.2(23932554_23944177)x1 |
| Relapse (+225d) | 16 | 16 | 4118206 | 4148313 | 0.99 | deletion | NA | 0.29 | 6848 | 0 | ADCY9 | - | 193 | ogm[GRCh37] 16p13.3(4118206_4148312)x1 |
| Relapse (+225d) | 18 | 18 | 7914268 | 8116237 | 0.99 | deletion | NA | 0.26 | 197730 | 0 | PTPRM | - | 100 | ogm[GRCh37] 18p11.23(7914268_8116237)x1 |
| Relapse (+225d) | 20 | 20 | 44203215 | 44209690 | 0.99 | deletion | NA | 0.19 | 3185 | 0 | WFDC8 | - | 131 | ogm[GRCh37] 20q13.12(44203215_44209690)x1 |
| Relapse (+225d) | 20 | 20 | 44336706 | 44386740 | 0.99 | deletion | NA | 0.25 | 30747 | 0.6 | WFDC13;SPINT4 | - | 85 | ogm[GRCh37] 20q13.12(44336706_44386740)x1 |
| Relapse (+225d) | 21 | 21 | 47038229 | 47058698 | 0.99 | deletion | NA | 0.34 | 6482 | 0 | - | - | 195 | ogm[GRCh37] 21q22.3(47038229_47058698)x1 |
| Relapse (+225d) | 23 | 23 | 113347567 | 113361950 | 0.99 | deletion | NA | 0.3 | 7225 | 0.6 | - | - | 232 | ogm[GRCh37] 23q23(113347567_113361949)x1 |
| Relapse (+225d) | 1 | 1 | 225388244 | 225394366 | 0.99 | insertion | NA | 0.4 | 6220 | 0 | DNAH14 | - | 415 | ogm[GRCh37] ins(1;?)(q42.12;?) |
| Relapse (+225d) | 1 | 1 | 23152779 | 23164560 | 0.99 | insertion | NA | 0.22 | 6186 | 0.6 | EPHB2 | - | 119 | ogm[GRCh37] ins(1;?)(p36.12;?) |
| Relapse (+225d) | 2 | 2 | 234232954 | 234236530 | 0.99 | insertion | NA | 0.32 | 10610 | 0 | SAG | - | 275 | ogm[GRCh37] ins(2;?)(q37.1;?) |

| | | | | | | | | | | | | | | |
|---|---|---|---|---|---|---|---|---|---|---|---|---|---|---|
| Relapse (+225d) | 3 | 3 | 139131709 | 139137532 | 0.99 | insertion | NA | 0.31 | 6061 | 0.6 | - | - | 341 | ogm[GRCh37] ins(3;?)(q23;?) |
| Relapse (+225d) | 3 | 3 | 195820356 | 195839697 | 0.99 | insertion | NA | 0.59 | 4531 | 0.6 | - | - | 300 | ogm[GRCh37] ins(3;?)(q29;?) |
| Relapse (+225d) | 3 | 3 | 195820356 | 195840292 | 0.99 | insertion | NA | 0.59 | 3967 | 0.6 | - | - | 33 | ogm[GRCh37] ins(3;?)(q29;?) |
| Relapse (+225d) | 4 | 4 | 120735721 | 120744497 | 0.99 | insertion | NA | 0.27 | 6100 | 0.6 | - | - | 237 | ogm[GRCh37] ins(4;?)(q26;?) |
| Relapse (+225d) | 5 | 5 | 1837283 | 1872413 | 0.99 | insertion | NA | 0.5 | 4935 | 0.6 | - | - | 171 | ogm[GRCh37] ins(5;?)(p15.33;?) |
| Relapse (+225d) | 5 | 5 | 33059463 | 33064516 | 0.99 | insertion | NA | 0.27 | 6463 | 0 | - | - | 253 | ogm[GRCh37] ins(5;?)(p13.3;?) |
| Relapse (+225d) | 5 | 5 | 527789 | 574944 | 0.99 | insertion | NA | 0.25 | 3354 | 0.6 | MIR4456 | - | 51 | ogm[GRCh37] ins(5;?)(p15.33;?) |
| Relapse (+225d) | 6 | 6 | 30435443 | 30440786 | 0.99 | insertion | NA | 0.46 | 4841 | 0 | - | - | 151 | ogm[GRCh37] ins(6;?)(p21.33;?) |
| Relapse (+225d) | 6 | 6 | 31433503 | 31443800 | 0.99 | insertion | NA | 0.28 | 94426 | 0 | HCP5;HCG26 | - | 89 | ogm[GRCh37] ins(6;?)(p21.33;?) |
| Relapse (+225d) | 6 | 6 | 40586940 | 40591956 | 0.99 | insertion | NA | 0.28 | 6148 | 0 | - | - | 260 | ogm[GRCh37] ins(6;?)(p21.1;?) |
| Relapse (+225d) | 8 | 8 | 7051958 | 7069891 | 0.99 | insertion | NA | 0.12 | 5875 | 0.6 | - | - | 26 | ogm[GRCh37] ins(8;?)(p23.1;?) |
| Relapse (+225d) | 10 | 10 | 1272968 | 1284604 | 0.99 | insertion | NA | 0.36 | 76579 | 0 | ADARB2 | - | 83 | ogm[GRCh37] ins(10;?)(p15.3;?) |
| Relapse (+225d) | 10 | 10 | 18896114 | 18916103 | 0.99 | insertion | NA | 0.4 | 10060 | 0 | NSUN6 | - | 293 | ogm[GRCh37] ins(10;?)(p12.31;?) |
| Relapse (+225d) | 11 | 11 | 43151429 | 43153757 | 0.99 | insertion | NA | 0.23 | 6190 | 0 | - | - | 122 | ogm[GRCh37] ins(11;?)(p12;?) |
| Relapse (+225d) | 13 | 13 | 113309756 | 113329149 | 0.99 | insertion | NA | 0.34 | 16528 | 0 | C13orf35 | - | 136 | ogm[GRCh37] ins(13;?)(q34;?) |
| Relapse (+225d) | 13 | 13 | 90309287 | 90323108 | 0.99 | insertion | NA | 0.73 | 4385 | 0.6 | - | - | 187 | ogm[GRCh37] ins(13;?)(q31.3;?) |
| Relapse (+225d) | 1 | 1 | 155349797 | 155349797 | 1 | inversion | NA | 0.2 | -1 | 0.6 | ASH1L | - | 26 | ogm[GRCh37] inv(1)(q22q22) |
| Relapse (+225d) | 1 | -1 | 155278172 | -1 | 1 | inversion_partial | NA | 0.2 | -1 | -1 | - | - | -1 | ogm[GRCh37] inv(1)(q22q22) |
| Relapse (+225d) | 1 | 1 | 155349797 | 155349797 | 1 | inversion | NA | 0.2 | -1 | 0.6 | ASH1L | - | 84 | ogm[GRCh37] inv(1)(q22q22) |
| Relapse (+225d) | 1 | -1 | 155279255 | -1 | 1 | inversion_partial | NA | 0.2 | -1 | -1 | - | - | -1 | ogm[GRCh37] inv(1)(q22q22) |
| Relapse (+225d) | 3 | 3 | 16752942 | 17864305 | 1 | inversion | NA | 0.01 | -1 | 0 | PLCL2;TBC1D5;TRNA_Pseudo | - | 5 | ogm[GRCh37] inv(3)(p24.3p24.3) |

| | | | | | | | | | | | | | | |
|---|---|---|---|---|---|---|---|---|---|---|---|---|---|---|
| Relapse (+225d) | 3 | -1 | 17946841 | -1 | 1 | inversion_partial | NA | 0.01 | -1 | -1 | - | - | -1 | ogm[GRCh37] inv(3)(p24.3p24.3) |
| Relapse (+225d) | 3 | 3 | 5935872 | 6573492 | 0.96 | inversion | NA | 0.14 | -1 | 0 | AF279782 | - | 35 | ogm[GRCh37] inv(3)(p26.1p26.1) |
| Relapse (+225d) | 3 | -1 | 5856855 | -1 | 0.96 | inversion_partial | NA | 0.14 | -1 | -1 | - | - | -1 | ogm[GRCh37] inv(3)(p26.1p26.1) |
| Relapse (+225d) | 5 | 5 | 115945474 | 115956150 | 1 | inversion | NA | 0.21 | -1 | 0 | - | - | 60 | ogm[GRCh37] inv(5)(q23.1q23.1) |
| Relapse (+225d) | 5 | -1 | 116088873 | -1 | 1 | inversion_partial | NA | 0.21 | -1 | -1 | - | - | -1 | ogm[GRCh37] inv(5)(q23.1q23.1) |
| Relapse (+225d) | 5 | 5 | 115959912 | 115969748 | 1 | inversion | NA | 0.21 | -1 | 0 | - | - | 7 | ogm[GRCh37] inv(5)(q23.1q23.1) |
| Relapse (+225d) | 5 | -1 | 116077394 | -1 | 1 | inversion_partial | NA | 0.21 | -1 | -1 | - | - | -1 | ogm[GRCh37] inv(5)(q23.1q23.1) |
| Relapse (+225d) | 10 | 10 | 64177328 | 64462742 | 1 | inversion | NA | 0.07 | -1 | 0 | ZNF365 | - | 10 | ogm[GRCh37] inv(10)(q21.2q21.2) |
| Relapse (+225d) | 10 | -1 | 64004769 | -1 | 1 | inversion_partial | NA | 0.07 | -1 | -1 | - | - | -1 | ogm[GRCh37] inv(10)(q21.2q21.2) |
| Relapse (+225d) | 10 | 10 | 66823948 | 70495589 | 0.99 | inversion | NA | 0.3 | -1 | 0 | CTNNA3;SnoU40;LRRTM3;HI650153;DNAJC12;U6;SIRT1;HERC4;MYPN;7SK;ATOH7;PBLD;HNRNPH3;RUFY2;DNA2;SLC25A16;TET1;CCAR1 | - | 153 | ogm[GRCh37] inv(10)(q21.3q21.3) |
| Relapse (+225d) | 10 | -1 | 70700810 | -1 | 0.99 | inversion_partial | NA | 0.3 | -1 | -1 | - | - | -1 | ogm[GRCh37] inv(10)(q21.3q21.3) |
| Relapse (+225d) | 10 | 10 | 70495589 | 70736620 | 0.75 | inversion | NA | 0.19 | -1 | 0 | CCAR1;SNORD98;STOX1;DDX50;DDX21 | - | 104 | ogm[GRCh37] inv(10)(q21.3q22.1) |
| Relapse (+225d) | 10 | -1 | 70297175 | -1 | 0.75 | inversion_partial | NA | 0.19 | -1 | -1 | - | - | -1 | ogm[GRCh37] inv(10)(q21.3q22.1) |
| Relapse (+225d) | 10 | 10 | 70597919 | 70740707 | 1 | inversion | NA | 0.19 | -1 | 0 | STOX1;DDX50;DDX21 | - | 7 | ogm[GRCh37] inv(10)(q21.3q22.1) |
| Relapse (+225d) | 10 | -1 | 70436832 | -1 | 1 | inversion_partial | NA | 0.19 | -1 | -1 | - | - | -1 | ogm[GRCh37] inv(10)(q21.3q22.1) |
| Relapse (+225d) | 10 | 10 | 70880833 | 70892163 | 1 | inversion | NA | 0.02 | -1 | 0 | VPS26A | - | 8 | ogm[GRCh37] inv(10)(q22.1q22.1) |
| Relapse (+225d) | 10 | -1 | 70769692 | -1 | 1 | inversion_partial | NA | 0.02 | -1 | -1 | - | - | -1 | ogm[GRCh37] inv(10)(q22.1q22.1) |
| Relapse (+225d) | 1 | 11 | 155384865 | 3755020 | 0.27 | translocation_interchr | -/- | 0.16 | -1 | 0 | ASH1L;NUP98 | ASH1L-NUP98 | 57 | ogm[GRCh37] t(1;11)(q22;p15.4) |
| Relapse (+225d) | 1 | 9 | 156684968 | 36297734 | 0.96 | translocation_interchr | +/- | 0.04 | -1 | 0 | - | - | 8 | ogm[GRCh37] t(1;9)(q23.1;p13.3) |
| Relapse (+225d) | 3 | 10 | 10273471 | 71641510 | 0.38 | translocation_interchr | +/+ | 0.02 | -1 | 0 | IRAK2;COL13A1 | IRAK2-COL13A1 | 8 | ogm[GRCh37] t(3;10)(p25.3;q22.1) |
| Relapse (+225d) | 3 | 10 | 14768714 | 70495589 | 1 | translocation_interchr | +/- | 0.15 | -1 | 0 | C3orf20;CCAR1 | C3orf20-CCAR1 | 43 | ogm[GRCh37] t(3;10)(p25.1;q21.3) |

| | | | | | | | | | | | | | | |
|---|---|---|---|---|---|---|---|---|---|---|---|---|---|---|
| Relapse (+225d) | 3 | 10 | 15610571 | 64319172 | 0.96 | translocation_interchr | +/+ | 0.02 | -1 | 0 | HACL1;ZNF365 | HACL1-ZNF365 | 5 | ogm[GRCh37] t(3;10)(p25.1;q21.2) |
| Relapse (+225d) | 3 | 10 | 16344791 | 72152951 | 1 | translocation_interchr | +/+ | 0.41 | -1 | 0 | OXNAD1 | - | 157 | ogm[GRCh37] t(3;10)(p25.1;q22.1) |
| Relapse (+225d) | 3 | 21 | 16778711 | 30261569 | 0.73 | translocation_interchr | +/+ | 0.02 | -1 | 0 | - | - | 5 | ogm[GRCh37] t(3;21)(p24.3;q21.3) |
| Relapse (+225d) | 3 | 10 | 17922015 | 64929786 | 0.68 | translocation_interchr | -/+ | 0.01 | -1 | 0 | JMJD1C | - | 5 | ogm[GRCh37] t(3;10)(p24.3;q21.3) |
| Relapse (+225d) | 3 | 10 | 17933829 | 64206980 | 0.97 | translocation_interchr | +/+ | 0.02 | -1 | 0 | ZNF365 | - | 5 | ogm[GRCh37] t(3;10)(p24.3;q21.2) |
| Relapse (+225d) | 3 | 10 | 18210816 | 64882177 | 0.86 | translocation_interchr | +/- | 0.02 | -1 | 0 | LOC339862 | - | 8 | ogm[GRCh37] t(3;10)(p24.3;q21.3) |
| Relapse (+225d) | 3 | 21 | 18733184 | 42711338 | 0.33 | translocation_interchr | +/+ | 0.03 | -1 | 0 | FAM3B | - | 7 | ogm[GRCh37] t(3;21)(p24.3;q22.3) |
| Relapse (+225d) | 3 | 10 | 7650922 | 72512994 | 0.98 | translocation_interchr | +/- | 0.08 | -1 | 0 | GRM7;ADAMTS14 | GRM7-ADAMTS14 | 22 | ogm[GRCh37] t(3;10)(p26.1;q22.1) |
| Relapse (+225d) | 3 | 10 | 8316163 | 64935875 | 0.75 | translocation_interchr | +/+ | 0.02 | -1 | 0 | LMCD1-AS1;JMJD1C | LMCD1-AS1-JMJD1C | 7 | ogm[GRCh37] t(3;10)(p26.1;q21.3) |
| Relapse (+225d) | 3 | 10 | 8383755 | 71718153 | 1 | translocation_interchr | +/+ | 0.27 | -1 | 0 | LMCD1-AS1;COL13A1 | LMCD1-AS1-COL13A1 | 126 | ogm[GRCh37] t(3;10)(p26.1;q22.1) |
| Relapse (+225d) | 3 | 10 | 86013858 | 63895519 | 0.97 | translocation_interchr | +/+ | 0.14 | -1 | 0 | CADM2 | - | 63 | ogm[GRCh37] t(3;10)(p12.1;q21.2) |
| Relapse (+225d) | 3 | 10 | 86136385 | 71272364 | 1 | translocation_interchr | +/+ | 0.17 | -1 | 0 | - | - | 51 | ogm[GRCh37] t(3;10)(p12.1;q22.1) |
| Relapse (+225d) | 3 | 10 | 86634401 | 63706716 | 1 | translocation_interchr | +/+ | 0.3 | -1 | 0 | ARID5B | - | 126 | ogm[GRCh37] t(3;10)(p12.1;q21.2) |
| Relapse (+225d) | 3 | 10 | 9689847 | 72541265 | 0.98 | translocation_interchr | +/+ | 0.28 | -1 | 0 | TBATA | - | 103 | ogm[GRCh37] t(3;10)(p25.3;q22.1) |
| Relapse (+225d) | 5 | 10 | 132131355 | 75647791 | 1 | translocation_interchr | +/+ | 0.25 | -1 | 0 | - | - | 85 | ogm[GRCh37] t(5;10)(q31.1;q22.2) |
| Relapse (+225d) | 7 | 17 | 54524974 | 35512065 | 0.53 | translocation_interchr | +/+ | 0.02 | -1 | 0 | ACACA | - | 7 | ogm[GRCh37] t(7;17)(p11.2;q12) |
| Relapse (+225d) | 7 | 10 | 56030988 | 71551093 | 0.48 | translocation_interchr | -/+ | 0.02 | -1 | 0 | - | - | 6 | ogm[GRCh37] t(7;10)(p11.2;q22.1) |
| Relapse (+225d) | 10 | 21 | 72180437 | 34005936 | 0.81 | translocation_interchr | +/+ | 0.06 | -1 | 0 | EIF4EBP2;SYNJ1 | EIF4EBP2-SYNJ1 | 12 | ogm[GRCh37] t(10;21)(q22.1;q22.11) |
| Relapse (+225d) | 11 | 21 | 21948118 | 17221321 | 0.74 | translocation_interchr | +/- | 0.03 | -1 | 0 | USP25 | - | 6 | ogm[GRCh37] t(11;21)(p14.3;q21.1) |
| Relapse (+225d) | 3 | 3 | 11703830 | 141424983 | 0.99 | translocation_intrachr | -/+ | 0.2 | -1 | 0 | - | - | 82 | ogm[GRCh37] fus(3;3)(p25.3;q23) |

| | | | | | | | | | | | | | | |
|---|---|---|---|---|---|---|---|---|---|---|---|---|---|---|
| Relapse (+225d) | 3 | 3 | 17664324 | 87820862 | 0.76 | translocation_intrachr | +/+ | 0.01 | -1 | 0 | TBC1D5 | - | 5 | ogm[GRCh37] fus(3;3)(p24.3;p11.2) |
| Relapse (+225d) | 3 | 3 | 18334993 | 158408263 | 0.96 | translocation_intrachr | -/+ | 0.02 | -1 | 0 | GFM1 | - | 14 | ogm[GRCh37] fus(3;3)(p24.3;q25.32 ) |
| Relapse (+225d) | 3 | 3 | 19043437 | 85941272 | 1 | translocation_intrachr | +/+ | 0.36 | -1 | 0 | CADM2 | - | 131 | ogm[GRCh37] fus(3;3)(p24.3;p12.1) |
| Relapse (+225d) | 3 | 3 | 5935872 | 87094899 | 0.82 | translocation_intrachr | +/+ | 0.17 | -1 | 0 | - | - | 56 | ogm[GRCh37] fus(3;3)(p26.1;p12.1) |
| Relapse (+225d) | 3 | 3 | 5935872 | 87109137 | 0.76 | translocation_intrachr | +/+ | 0.17 | -1 | 0 | - | - | 56 | ogm[GRCh37] fus(3;3)(p26.1;p12.1) |
| Relapse (+225d) | 3 | 3 | 7921134 | 84406015 | 0.34 | translocation_intrachr | +/+ | 0.06 | -1 | 0 | - | - | 45 | ogm[GRCh37] fus(3;3)(p26.1;p12.1) |
| Relapse (+225d) | 3 | 3 | 7921824 | 15366443 | 0.85 | translocation_intrachr | -/+ | 0.03 | -1 | 0 | SH3BP5 | - | 29 | ogm[GRCh37] fus(3;3)(p26.1;p25.1) |
| Relapse (+225d) | 3 | 3 | 8161284 | 18717679 | 0.71 | translocation_intrachr | +/+ | 0.02 | -1 | 0 | - | - | 7 | ogm[GRCh37] fus(3;3)(p26.1;p24.3) |
| Relapse (+225d) | 7 | 7 | 49327245 | 56028783 | 0.54 | translocation_intrachr | +/- | 0.02 | -1 | 0 | - | - | 7 | ogm[GRCh37] fus(7;7)(p12.2;p11.2) |
| Relapse (+225d) | 21 | 21 | 24252871 | 42525873 | 0.24 | translocation_intrachr | -/+ | 0.01 | -1 | 0 | - | - | 5 | ogm[GRCh37] fus(21;21)(q21.2;q22. 2) |
| Relapse (+225d) | 21 | 21 | 24495432 | 29856259 | 0.18 | translocation_intrachr | -/+ | 0.03 | -1 | 0 | - | - | 6 | ogm[GRCh37] fus(21;21)(q21.2;q21. 3) |
| Relapse (+225d) | 6 | 6 | 115447157 | 115495071 | -1 | duplication | NA | 0.93 | 47915 | 0 | - | - | 81 | ogm[GRCh37] dup(6)(q22.1q22.1) |
| Relapse (+225d) | 6 | 6 | 115449485 | 115556170 | -1 | duplication | NA | 0.2 | 106686 | 0 | - | - | 79 | ogm[GRCh37] dup(6)(q22.1q22.1) |
| Relapse (+225d) | 6 | 6 | 31358385 | 31443800 | -1 | duplication | NA | 0.52 | 85416 | 0 | MICA;HCP5;HCG26 | - | 70 | ogm[GRCh37] dup(6)(p21.33p21.33 ) |
| Relapse (+225d) | 7 | 7 | 64992019 | 65031612 | -1 | duplication | NA | 0.38 | 39594 | 0 | - | - | 88 | ogm[GRCh37] dup(7)(q11.21q11.21 ) |
| Relapse (+225d) | 5 | 5 | 115956150 | 116077394 | -1 | duplication_inverted | NA | 0.33 | 121245 | 0 | Mir_633 | - | 39 | ogm[GRCh37] dup(5)(q23.1q23.1) |
| Relapse (+225d) | 5 | 5 | 115956150 | 116088873 | -1 | duplication_inverted | NA | 0.33 | 132724 | 0 | Mir_633 | - | 74 | ogm[GRCh37] dup(5)(q23.1q23.1) |

**Supplementary Table 3:** Optical Genome Mapping - Rare Variant Pipeline Rare – Confident Copy Number Variants

| Time point of analysis (Day) | Chr | Reference Start | Reference End | Size in bases | Type | fractional Copy Number | Copy Number | Confidence | VAF |
|---|---|---|---|---|---|---|---|---|---|
| AML diagnosis (0d) | 1 | 246656863 | 249237532 | 2580670 | gain | 2.436 | 2 | 0.999999993 | 0.217879327 |
| AML diagnosis (0d) | 3 | 61829 | 6566166 | 6504338 | loss | 1.139 | 1 | 1 | 0.430326857 |
| AML diagnosis (0d) | 3 | 6997453 | 8342330 | 1344878 | loss | 1.401 | 1 | 1 | 0.299701917 |
| AML diagnosis (0d) | 3 | 8348572 | 9672818 | 1324247 | loss | 1.136 | 1 | 1 | 0.431986042 |
| AML diagnosis (0d) | 3 | 9680389 | 14839124 | 5158736 | loss | 1.460 | 1 | 1 | 0.269786568 |
| AML diagnosis (0d) | 3 | 14853297 | 16361890 | 1508594 | loss | 1.331 | 1 | 1 | 0.334635466 |
| AML diagnosis (0d) | 3 | 19043437 | 85935616 | 66892180 | loss | 1.116 | 1 | 1 | 0.442248822 |
| AML diagnosis (0d) | 3 | 87777096 | 88584653 | 807558 | gain | 2.458 | 2 | 0.999995058 | 0.229211347 |
| AML diagnosis (0d) | 3 | 89064454 | 90470323 | 1405870 | loss | 1.463 | 1 | 0.999998782 | 0.268466665 |
| AML diagnosis (0d) | 3 | 93508428 | 168821068 | 75312641 | loss | 1.564 | 2 | 1 | 0.218248752 |
| AML diagnosis (0d) | 5 | 132131355 | 180899715 | 48768361 | loss | 1.111 | 1 | 1 | 0.444318206 |
| AML diagnosis (0d) | 10 | 17448661 | 17962252 | 513592 | gain | 2.494 | 2 | 0.999999309 | 0.246993159 |
| AML diagnosis (0d) | 10 | 42408726 | 46418799 | 4010074 | loss | 1.131 | 1 | 1 | 0.434481743 |
| AML diagnosis (0d) | 10 | 48107282 | 49071953 | 964672 | loss | 1.020 | 1 | 1 | 0.490234346 |
| AML diagnosis (0d) | 10 | 49084367 | 51125780 | 2041414 | loss | 1.089 | 1 | 1 | 0.455680521 |
| AML diagnosis (0d) | 10 | 51398298 | 62428151 | 11029854 | loss | 1.096 | 1 | 1 | 0.452075057 |
| AML diagnosis (0d) | 10 | 66822698 | 70285006 | 3462309 | loss | 1.095 | 1 | 1 | 0.452400843 |
| AML diagnosis (0d) | 10 | 72462296 | 75699352 | 3237057 | loss | 1.096 | 1 | 1 | 0.452090902 |
| AML diagnosis (0d) | 11 | 125802530 | 130123237 | 4320708 | gain | 2.327 | 2 | 0.999976263 | 0.163386463 |
| AML diagnosis (0d) | 16 | 2263650 | 21369108 | 19105459 | loss | 1.674 | 2 | 0.999985365 | 0.163087363 |
| AML diagnosis (0d) | 16 | 22822268 | 32019651 | 9197384 | loss | 1.688 | 2 | 0.999980906 | 0.156072851 |
| AML diagnosis (0d) | 16 | 46438848 | 85148570 | 38709723 | loss | 1.679 | 2 | 0.999996089 | 0.160730158 |
| Relapse (+225d) | 1 | 155343758 | 158120830 | 2777073 | gain | 2.403 | 2 | 0.99999999 | 0.201653839 |
| Relapse (+225d) | 1 | 158125568 | 158955052 | 829485 | gain | 2.677 | 3 | 1 | 0.338344699 |
| Relapse (+225d) | 1 | 158956531 | 205916326 | 46959796 | gain | 2.496 | 2 | 1 | 0.248020756 |
| Relapse (+225d) | 1 | 205917505 | 234072600 | 28155096 | gain | 2.461 | 2 | 1 | 0.230680905 |
| Relapse (+225d) | 1 | 234074062 | 235161536 | 1087475 | gain | 2.650 | 3 | 1 | 0.325129032 |
| Relapse (+225d) | 1 | 235165389 | 245865547 | 10700159 | gain | 2.474 | 2 | 1 | 0.236758875 |
| Relapse (+225d) | 1 | 245871339 | 249237532 | 3366194 | gain | 2.617 | 3 | 1 | 0.308515864 |
| Relapse (+225d) | 3 | 61829 | 6647413 | 6585585 | loss | 1.532 | 2 | 1 | 0.233851276 |
| Relapse (+225d) | 3 | 8359889 | 9690592 | 1330704 | loss | 1.498 | 1 | 1 | 0.250796699 |
| Relapse (+225d) | 3 | 16384192 | 19041596 | 2657405 | gain | 2.443 | 2 | 1 | 0.221736922 |
| Relapse (+225d) | 3 | 19043437 | 61555309 | 42511873 | loss | 1.499 | 1 | 1 | 0.250595064 |
| Relapse (+225d) | 3 | 61559498 | 68436384 | 6876887 | loss | 1.441 | 1 | 1 | 0.279622487 |
| Relapse (+225d) | 3 | 68439578 | 85935616 | 17496039 | loss | 1.525 | 2 | 1 | 0.237250689 |
| Relapse (+225d) | 3 | 141393291 | 161042132 | 19648842 | gain | 2.391 | 2 | 0.999999685 | 0.195737799 |

| | | | | | | | | | |
|---|---|---|---|---|---|---|---|---|---|
| Relapse (+225d) | 3 | 161046813 | 197957467 | 36910655 | gain | 2.477 | 2 | 1 | 0.238472357 |
| Relapse (+225d) | 5 | 132065863 | 180899715 | 48833853 | loss | 1.494 | 1 | 1 | 0.252900709 |
| Relapse (+225d) | 10 | 517019 | 17962252 | 17445234 | gain | 2.451 | 2 | 1 | 0.225701756 |
| Relapse (+225d) | 10 | 17963081 | 39074032 | 21110952 | gain | 2.446 | 2 | 0.999999999 | 0.223194819 |
| Relapse (+225d) | 10 | 42907613 | 46418799 | 3511187 | loss | 1.472 | 1 | 1 | 0.264187944 |
| Relapse (+225d) | 10 | 48107282 | 48747765 | 640484 | loss | 1.409 | 1 | 1 | 0.295625551 |
| Relapse (+225d) | 10 | 49084367 | 51125780 | 2041414 | loss | 1.452 | 1 | 1 | 0.274092766 |
| Relapse (+225d) | 10 | 51398298 | 62428151 | 11029854 | loss | 1.497 | 1 | 1 | 0.251286239 |
| Relapse (+225d) | 10 | 62429156 | 63388827 | 959672 | gain | 2.472 | 2 | 0.999999997 | 0.235993032 |
| Relapse (+225d) | 10 | 63722712 | 65892309 | 2169598 | gain | 2.562 | 3 | 1 | 0.281143168 |
| Relapse (+225d) | 10 | 66791150 | 70285006 | 3493857 | loss | 1.532 | 2 | 1 | 0.233772502 |
| Relapse (+225d) | 10 | 72504636 | 75695386 | 3190751 | loss | 1.464 | 1 | 1 | 0.268229864 |
| Relapse (+225d) | 21 | 14755232 | 47282074 | 32526843 | gain | 2.446 | 2 | 1 | 0.223132469 |
| Relapse (+225d) | 22 | 16695297 | 17500113 | 804817 | gain | 2.996 | 3 | 1 | 0.498021235 |
| Relapse (+225d) | 22 | 17509712 | 18645711 | 1136000 | gain | 2.691 | 3 | 1 | 0.345268 |
| Relapse (+225d) | 22 | 18864171 | 20356873 | 1492703 | gain | 2.885 | 3 | 1 | 0.442335644 |
| Relapse (+225d) | 22 | 20625703 | 48840122 | 28214420 | gain | 2.816 | 3 | 1 | 0.408110605 |

## **Supplementary Table 4:** Optical Genome Mapping - Rare Variant Pipeline – Confident Aneuploidy Calls

| **Time point** | **Chr** | **Aneuploidy type** | **Fraction of complete chromosome length** | **Confidence** | **Fractional Copy Number** |
|---|---|---|---|---|---|
| AML diagnosis (0d) | 3 | loss | 0,8204514067 | 1 | 1,114170659 |
| AML diagnosis (0d) | 16 | loss | 0,8778554944 | 0,9999999744 | 1,633344541 |
| Relapse (+225d) | 21 | gain | 0,936071812 | 1 | 2,444302749 |
| Relapse (+225d) | 22 | gain | 0,965232492 | 1 | 2,877173468 |
